# Optic ataxia in patients with thalamic lesions

**DOI:** 10.1093/braincomms/fcaf359

**Published:** 2025-09-13

**Authors:** Melanie Wilke, Hanna J Eisenberg, Carsten Schmidt-Samoa, Shirin Mahdavi, Igor Kagan, Peter Dechent, Jan Liman, Hans-Otto Karnath, Mathias Bähr

**Affiliations:** Department of Cognitive Neurology, Heart & Brain Center, University Medical Center Goettingen, Goettingen 37075, Germany; Cognitive Neurology Group, Cognitive Neuroscience Laboratory, German Primate Center – Leibniz Institute for Primate Research, Goettingen 37075, Germany; Clinic of Neurology, University Medical Center Goettingen, Goettingen 37075, Germany; Department of Cognitive Neurology, Heart & Brain Center, University Medical Center Goettingen, Goettingen 37075, Germany; Department of Cognitive Neurology, Heart & Brain Center, University Medical Center Goettingen, Goettingen 37075, Germany; Department of Cognitive Neurology, Heart & Brain Center, University Medical Center Goettingen, Goettingen 37075, Germany; Decision and Awareness Group, Cognitive Neuroscience Laboratory, German Primate Center—Leibniz Institute for Primate Research, Goettingen 37075, Germany; Department of Cognitive Neurology, Heart & Brain Center, University Medical Center Goettingen, Goettingen 37075, Germany; Clinic of Neurology, University Medical Center Goettingen, Goettingen 37075, Germany; Department of Neurology, University Medicine Center Nuremberg, Paracelsus Private University, Nuremberg 90471, Germany; Center of Neurology, Division of Neuropsychology, Hertie-Institute for Clinical Brain Research, University of Tuebingen, Tuebingen 72076, Germany; Clinic of Neurology, University Medical Center Goettingen, Goettingen 37075, Germany

**Keywords:** pulvinar, central thalamus, mediodorsal thalamus, reaching, visuomotor

## Abstract

Lesions in the parietal cortex can strongly impair visually guided reach-grasping behaviour. A specific reaching deficit termed ‘optic ataxia’ (OA) occurs when eye and hand position are dissociated. OA has typically been studied in patients with lesions in the parietal cortex, neglecting potential thalamic contributions. Here, we examined 28 acute stroke patients (age 58.9 ± 12.6 years) with circumscribed thalamic lesions for the presence of OA. We leveraged MRI-based lesion-symptom mapping to address the contributions of specific thalamic nuclei to visually guided reaching deficits under foveal and peripheral viewing conditions. Based on the cortical literature, we hypothesized that lesions in thalamic nuclei with strong connections to the inferior and superior parietal cortex, such as the ventrolateral nucleus and pulvinar might lead to OA. In comparison with age-matched healthy subjects (*n* = 40, age 60.6 ± 9.1 years), we identified five thalamic patients with OA, most pronounced for reaches with the contralesional hand into the contralesional space. While motor and grasping deficits and OA occurred frequently together, they did not always co-occur, and visual attention deficits could not account for the OA either. Comparing the lesion maps of patients with and without OA, the critical lesion site for OA was not restricted to one circumscribed thalamic region within the Morel atlas. Instead, it encompassed several medial and lateral nuclei within and around the internal medullary laminar complex. Interestingly, this region matches the so-called central thalamus, a functionally defined thalamic region that is considered a ‘higher-order’ nucleus complex. It receives afferent inputs from the cerebellum and brainstem regions and connects to fronto-parietal regions involved in eye movement control. Taken together, our results suggest the critical importance of thalamic nuclei for the spatial transformation from eye- into body-centred coordinates.

## Teaser

Wilke *et al*. show that damage to certain thalamic regions—beyond the parietal cortex—can cause optic ataxia, a reach–grasp disorder. Seen in stroke patients when they do not look directly at objects, it highlights the crucial role of the thalamus in translating peripheral visual input into coordinated motor actions.

## Graphical Abstract

**Graphical Abstract:**
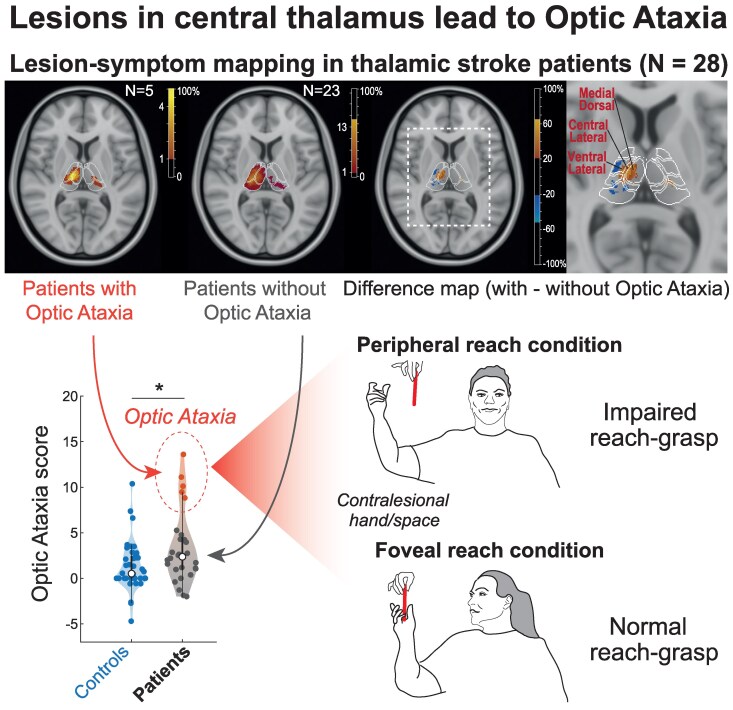

## Introduction

Optic ataxia (OA) is a specific type of visuomotor deficit related to the interaction with visual objects in foveal versus extrafoveal (i.e. peripheral) space. It is a compound syndrome that consists of misreaching into the visual periphery, difficulties in grasping objects and online correction of hand movements.^1,2^ The defining OA feature, however, is the difficulty to reach–grasp objects in peripheral space, i.e. when the gaze direction is dissociated from the reach goal.^3^ In unilateral lesion cases, reach errors in OA are most pronounced when patients use their contralesional hand to reach into contralesional space. Isolated OA in humans is rare, but patient cases with intact visual fields and preserved ocular and limb motor control have been described.^4^ Generally, reach–grasp functions are thought to rely on a distributed network of cortical regions such as motor, premotor, supplementary motor, superior parietal and dorsal occipital cortex as well as the cerebellum.^5,6^ Amongst those regions, OA has been specifically associated with lesions in the superior parietal lobule (SPL), intraparietal sulcus and the junction between the inferior parietal lobule (IPL) and superior occipital cortex.^4,7^ Since SPL and IPL are anatomically and functionally positioned between visual cortex and somatomotor-related prefrontal regions, they are thought to integrate visual and somatic information important for the control of movements.^8^ While the search for the neural mechanisms behind visually guided reaches primarily focused on cortical regions, there are several thalamic nuclei with strong connections to SPL/IPL. These thalamic connections are evidenced by anatomical tracer and microstimulation studies in macaques^8-10^ as well as by anatomical and functional connectivity studies in humans.^11,12^ Specifically, SPL and IPL share reciprocal connections with somatomotor-related thalamic nuclei such as the ventral lateral (VL), ventral posterior lateral (VPL) and central lateral/centromedial nuclei (CL/CM), which also receive input from the cerebellum and basal ganglia.^13,14^ Clinically, lesions in those thalamic nuclei have been associated with motor symptoms such as ataxia, dystonia, tremor and motor learning deficits.^15-18^ In addition, SPL/IPL have strong connections with non-classical motor nuclei such as the lateral posterior nucleus and the dorsal pulvinar,^8,9,19^ which are thought to enable efficient information transfer across visual and fronto-parietal cortices.^20,21^

So far, it is unclear whether isolated thalamic lesions lead to OA. Studies of visually guided reach–grasp behaviour after thalamic lesions are exceedingly rare, and the human literature consists almost entirely of individual case studies. In one case study, OA signified by misreaches towards contralesional peripheral space was reported in a patient with left-sided lesions entailing the midbrain, cerebellum and thalamus.^22^ Another case study in a patient with bilateral medial pulvinar lesions reported abnormal reach–grasp behaviour. The reach–grasp deficits of this pulvinar patient differed from OA, however, as reach errors were not alleviated when he was allowed to look at the target.^23^ Two studies in non-human primates reported ‘OA’ following pharmacological inactivation or ablation of the pulvinar based on persistent misreaches and grasping deficits.^24,25^ Importantly, while imprecise reach-grasping is often considered to be part of the OA syndrome,^2^ the OA defining performance difference between foveal versus peripheral reaches was not tested in those monkey studies.

We here investigated whether thalamic lesions and, if so, which subnuclei lead to OA in humans. We tested 28 patients with circumscribed thalamic lesions and compared the lesion location in patients with and without OA. In contrast to the referred primate and human case studies, the current study investigated a reasonably sized group of thalamic lesion patients with stroke aetiology. In addition, we mapped the lesion locations on high-resolution structural MRI and the Morel atlas, which allows anatomical precision and overcomes the narrow focus on single nuclei such as the pulvinar that characterizes previous work. Based on the cortical literature, we hypothesized that lesions in thalamic nuclei with strong connections to SPL/IPL cortex and known participation in integrating visual and motor information, such as VL, pulvinar and LP might lead to OA. To our knowledge, this is the first patient population study that investigates the involvement of specific thalamic nuclei to visuomotor transformations required for gaze-dissociated reach movements.

## Materials and methods

### Study sample

We examined 28 patients with focal ischaemic (*n* = 26) or haemorrhagic (*n* = 2) thalamic lesions. The stroke lesion group included 21 males and 7 females; 14 patients had predominately left and 14 patients had predominately right thalamic lesions (Table 1). Of those, six patients had an additional thalamic lesion in the opposite hemisphere, but all had a clinically relevant larger lesion on one side, which we use as a reference for ipsi- versus contralesional assignments (Supplementary Tables 1 and 2). The most affected lesion territories were the medial, lateral and posterior thalamus (see section on lesion characteristics below). The mean age of the patients was 58.9 years (range: 24.8–80.5, SD = 12.6). Testing for OA was performed within 10 days after stroke onset (median: 4 days, range: 2–9 days). MRI data were acquired within 1 day of behavioural testing. Patients were recruited from acute stroke patients at the ward of the Clinic of Neurology at the University Medical Center Göttingen. Following initial assessment, including the clinical MRI or CT scan, patients with diffuse lesions, visual field deficits assessed by confrontation test, low vigilance or aphasia that would have interfered with understanding the task instructions were excluded. General exclusion criteria for patients and healthy controls (HCs) were psychiatric disorders, chronic substance abuse or inability to lie still in the MR scanner. Of the 28 patients, 19 patients received a cognitive screening with the Mini-Mental State Examination (MMSE)^26^ or the Montreal Cognitive Assessment (MoCA).^27^ The maximum score in each cognition test is 30, and patients achieved a mean score of 27.84 (range: 22–30) (Supplementary Table 3). In the patient population, 39.3% had somatosensory (including paraesthesia) deficits in the upper (contralesional) limb and 50% had a mild contralesional central hemiparesis of the upper limb (arm sinking in the holding test). The finger-to-nose test (FNT) was dysmetric in 14.3% of the patients. Light aphasia was present in 10.7% (Table 1). Signs of neglect, as defined by pathological results in two neglect tests, were present in one patient. Testing for spatial neglect was conducted with several paper-and-pencil tests of the German neglect battery^28^: (i) line bisection; (ii) line cancellation; (iii) star cancellation, the Apples test^29^ and a computerized Posner task^30,31^ (Supplementary Methods). We also recruited a group of 40 age-matched healthy participants (20 males/20 females). The mean age of the healthy subjects was 60.6 years (range: 40.4–81.9, SD = 9.1). A two-sided *t*-test confirmed that the age of patients and HCs did not differ [*t*(66) = 0.63, *P* = 0.53].

**Table 1:** Demographic information, lesion aetiology and clinical findings

	Measure	All thalamic patients	Left lesion subgroup	Right lesion subgroup
Number of patients	*n*	28	14	14
Right handers	*n*	27	14	13
Age	Years mean [range]	58.9 [24.8, 80.5]	53.4 [24.8, 70.9]	64.5 [50.7, 80.5]
Sex	*n* Sex males	21	12	9
Aetiology	*N* Infarct	26	12	14
Lesion size	Lesion size in mm^3^ [range]	1226.9 [170.8, 5712.9]	1527.5 [375.4, 5712.9]	1145.0 [170.8, 2751.8]
Lesion territory lateral	*n* Lateral	9	3	6
Lesion territory medial	*n* Medial	11	6	5
Lesion territory posterior	*n* Posterior	8	5	3
Time since lesion to experiment	Median days [range]	4.0 [2.0, 9.0]	4.0 [2.0, 9.0]	4.5 [2.0, 9.0]
Somatosensory upper contralesional	%present	39.3	35.7	42.9
Somatosensory lower contralesional	%present	25.0	28.6	21.4
Paresis upper contralesional	%present	50.0	50.0	50.0
Paresis lower contralesional	%present	32.1	35.7	28.6
Ataxia contralesional (finger-to-nose)	%present	14.3	14.3	14.3
Aphasia	%present	10.7	21.4	0
Neglect	%present	3.6	7.1	0

Subjects’ written informed consent was obtained according to the Declaration of Helsinki, and the study was approved by the medical ethics committee of the University Medical Center Goettingen, Germany. All patients signed an additional agreement to present their videos in scientific publications. This article follows the Strengthening the Reporting of Observational Studies in Epidemiology (STROBE) guidelines (https://www.strobe-statement.org/checklists/).^32^

### Assessment of stroke symptoms of interest

Neurological assessments of somatosensory, motor and aphasia symptoms were performed by clinicians on the ward and were later derived from the clinical documentation. Somatosensory symptoms included all documented abnormalities of touch, pain and temperature sensation on either arm/hand (‘upper’) and legs (‘lower’) and reported dysesthesia/paraesthesia, such as tingling and numbness. Motor evaluation included muscle tone and reflex status. Muscle strength was tested on arms (‘upper’) and legs (‘lower’) with the Medical Research Council (MRC) Scale for Muscle Strength and graded 0–5 (https://www.ukri.org/councils/mrc/facilities-and-resources/find-an-mrc-facility-or-resource/mrc-muscle-scale/). Ataxia refers to the dysmetria in the FNTs. Grasping performance was tested in our laboratory with small objects and assessed by scoring the movies, similar to our previous pulvinar inactivation/lesion experiments in monkeys and humans^23,25^ (Supplementary Methods, Supplementary Fig. 1).

### OA: main task description

**Figure 1:**
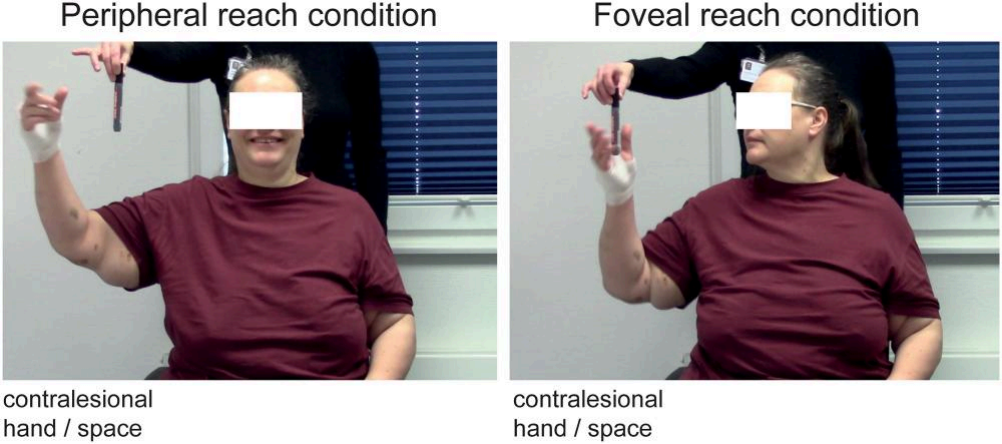
**OA task.** One examiner presented a pen to the participant while the other experimenter stood behind the video camera (not shown) and observed the performance of the patient including the eye position. The pen was always presented in the peripheral space relative to the participant but varied in height. Shown is a snapshot of an exemplary patient with OA from the current study. The patient had a lesion in the left thalamus that encompassed CM, VPL and pulvinar. The patient showed gross misreaches when the pen was presented in peripheral vision (eye fixation on the camera), while performing almost normal reaches under foveal vision (i.e. when allowed to orient eyes and head towards the object).

The OA task was adopted from published OA guidelines.^3^ The task consisted of grasping a pen being held into the peripheral visual field by the examiner standing behind the subject (Fig. 1), recorded by a second examiner using a digital camera. Participants were instructed by the experimenter on which hand to use. The pen was always presented in an upright orientation and peripheral with respect to the subject’s body, with varying height between the hand and elbow. Participants were either instructed to look at the pen as soon as they noticed it and then to reach for it (‘foveal reach’) or to keep fixating the camera while reaching for the pen (‘peripheral reach’). The examiner behind the camera controlled the performance, and the trial was repeated if hand or eye instructions were not met. In addition, trials were labelled as valid or invalid during the video-based offline scoring based on this criterion. The median of valid trials across participants and for each hand/field and viewing condition ranged between 11 and 16 trials.

### Analysis of the OA task

#### Reach error scores

Individual error rates were analysed by two independent raters who scored each trial offline from the recorded videos. Error categories were (in raw error points given by the rater): fluent (0), slowed/insecure (1), corrected during (2)/after first (3)/second reach (4)/failed to reach (5) (category 5 never occurred). Reach–grasp errors and OA scores were calculated similar to Borchers *et al*.^3^ First, a percentage mean error score was calculated for each of the eight (2 × 2 × 2, hand/space/gaze requirement) conditions using the formula mean error score = sum of trial error scores/(number of trials × 5)×100. The error scores thus range from 0 (no error in any trial) to 100%. Higher values reflect worse reach performance. As some of the literature discriminated between corrected and uncorrected reaches,^4,7^ we also calculated the percentage of corrected and uncorrected reach errors. To this end, we categorized the errors into ‘fluent’ (score: 0), ‘corrected’ (slowed, insecure and corrected during reach, score: 1–2) and ‘uncorrected’ (scores 3–5), i.e. when the hand stopped at the wrong position and was corrected only in later reaches.

#### OA scores

In order to derive OA scores, a difference value was calculated for each hand/field combination by subtracting the individual mean error score in the foveal condition from the score in the peripheral condition. By the subtraction of viewing conditions to yield OA scores, primary motor factors such as paresis or tremor should cancel out.^3^ All scores were converted into ipsilesional and contralesional hand/space. For example, in a patient with a lesion in the left thalamus, the ipsilesional hand/space would be left and the contralesional hand/space right. In order to match the HCs, it was determined that in 50% of the HCs data from the left hand/space should be used for ipsi hand/space. The HC group assignment to ipsi/contra was based on 100 000 permutations, which produced balanced mean and SD values for ipsi- and contralesional scores in the HCs.

### Structural brain imaging and lesion analysis

#### Anatomical MRI acquisition

MRI data were collected with a 3 T MR system (Magnetom TIM Trio, Siemens Healthineers, Erlangen, Germany, using a 32-channel phased-array head coil, or after scanner upgrade: Magnetom Prisma^fit^ using a 64-channel head coil). Three-dimensional (3D) anatomical datasets at 1 mm³ resolution were acquired in sagittal orientation with T1-weighting [turbo fast low angle shot, repetition time (TR): 2300 ms, inversion time (TI): 900 ms, echo time (TE): 2.96 ms (2.98 ms on Prisma^fit^), flip angle 9°] and with T2-weighting [fluid-attenuated inversion recovery (FLAIR), TR: 5000 ms, TI: 1800 ms, TE: 394 ms, integrated parallel acquisition technique: factor 2].

#### Anatomical lesion mapping

Lesions were manually segmented on FLAIR images using MRIcron. FSL 5.0.7 was used to coregister individual FLAIR images linearly to corresponding T1-weighted (T1w) images (FLIRT). T1w images were normalized to the MNI152 template [brain extraction (BET), 12-parameter affine (FLIRT) and non-linear registration (FNIRT), with lesions masked during registration]. The transformation matrices were applied to the whole-head T1w and FLAIR images and the lesion masks. Finally, the resulting images were upsampled to 0.5 mm isotropic resolution. A digitized version of the Morel atlas of the thalamus,^33,34^ registered to the 0.5 mm MNI152 template, allowed for visualization and analysis of the thalamic lesions with respect to thalamic nuclei (Supplementary Methods). For analysis in ipsi-/contralesional space, left thalamic lesions were mirrored along the midsagittal plane in MNI152 space. Individual scans and results of the lesion mapping are shown in Supplementary Fig. 2 and Table 1.

#### Demographic, clinical and behavioural statistical data analysis

In order to assess whether OA occurs in thalamic patients at the population level and to derive pathological scores, we performed group comparisons between age-matched HCs, thalamic stroke patients and the subgroups of these patients with and without OA. In order to test whether the reach errors and OA scores are normally distributed, a Shapiro–Wilk test was performed and showed that the distribution of those variables departed significantly from normality [all *P*-values < 0.05 in both, HC (*n* = 40) and patients (*n* = 28)]. Thus, group comparisons and *post hoc* tests were performed using the non-parametric Mann–Whitney U-test. For each statistical comparison, we report the exact *P*-value, the number of comparisons, the Bonferroni-corrected *α*-level, and whether the test was significant after Bonferroni correction. Since the reach error scores compared the two groups in four conditions, we assumed *α* = 0.05 as original and a corrected *α* (0.05/4 = 0.0125) for the *post hoc* comparisons. Since there were more than two conditions, for the reach error scores, we also calculated a mixed repeated-measures ANOVA with the between-subjects factor *GROUP* (healthy subjects versus patients) and two within-subject factors: *VIEWING CONDITION* (foveal versus peripheral) and *SIDE* (CH_CS versus IH–IS). In the description of the ANOVA results, we focus on the relevant *GROUP* and their interaction effects, i.e. *GROUP* × *VIEWING CONDITION*, *GROUP* × *SIDE* and *GROUP* × *VIEWING CONDITION* × *SIDE* effects. For the OA scores, which already represent the difference between viewing conditions, we adopted *α* = 0.05 as original, and a Bonferroni-corrected *α* (0.05/2 = 0.025) for the *post hoc* comparisons. All statistical tests were two-tailed. Behavioural data analysis was performed using custom written scripts in MATLAB R2019b (The MathWorks Inc., Natick, MA, USA) and with the software package SPSS (version 30.0.0; SPSS, Inc., Chicago, IL, https://www.ibm.com/spss).

#### Lesion characteristics and clinical profile of the patients

**Figure 2:**
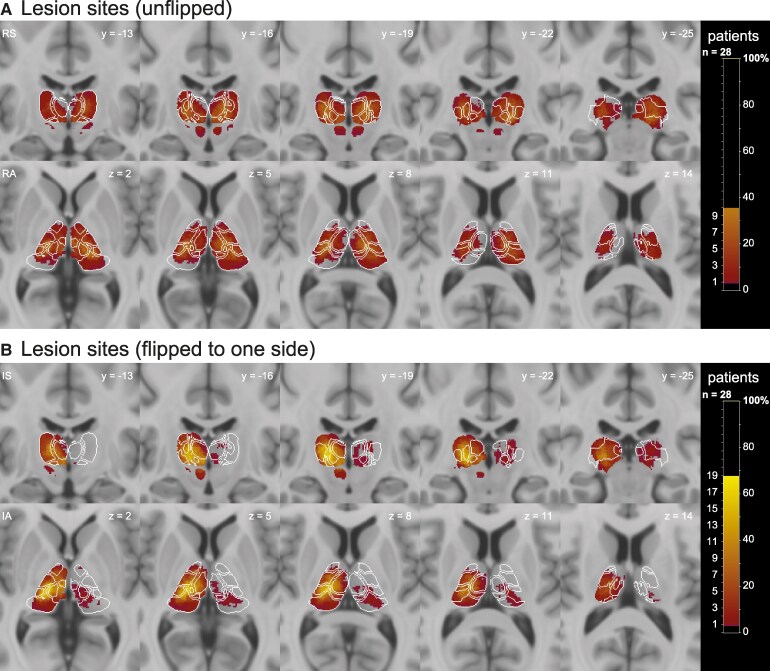
**Lesion frequency map of the thalamic lesions in the whole group of stroke patients (*n*_patients_  *=* 28).** (**A**) Lesion overlap maps (descriptive) as they appear in the left and right hemisphere (radiological convention). Percentage of overlapping lesions is illustrated by increasingly brighter colours representing increasing patient frequencies. (**B**) Lesions flipped so that the predominant lesion always appears in the right hemisphere. Coronal slices from anterior to posterior (left to right). Axial slices from inferior to superior. Lesions were manually segmented on FLAIR MR images using MRIcron and transformed into MNI space. The colour scale represents the number and percentage of patients with a lesion in a given voxel, with brighter colours representing higher lesion frequencies. Upper panels in **A** and **B** show the coronal, the lower panels the axial slices. In **A** and **B**, the numbers refer to the MNI coordinates of the *y*-axis (coronal slices) and *z*-axis (axial slices), respectively. R, right; S, superior; I, ipsilateral for flipped; A, anterior (e.g. RS is ‘right superior’). No additional statistics were applied.

Thalamic lesions centred in 9 patients on the medial thalamus, in 11 on the lateral and in 8 on the posterior thalamus (Supplementary Tables 1 and 2). From the medial group, which includes the intralaminar nuclei (ILN), CM (23 patients, 82.1%), CL (14 patients, 50%), parafascicular nucleus (13 patients, 46.4%) and the mediodorsal nucleus (MD) (11 patients, 39.3%) were the most frequently affected. From the lateral portion, the ventral posterior (VP) (20 patients, 71%), VL (15 patients, 53%) and the ventral medial (VM) nucleus were damaged in 11 patients (39%). From the posterior group, the pulvinar and the lateral posterior nucleus were damaged in 12 (43%) and 11 patients (39%), respectively. The majority of patients had unilateral lesions, while six patients also had some damage in the other hemisphere. Figure 2 shows the overall lesion pattern for the entire group.

## Results

**Figure 3:**
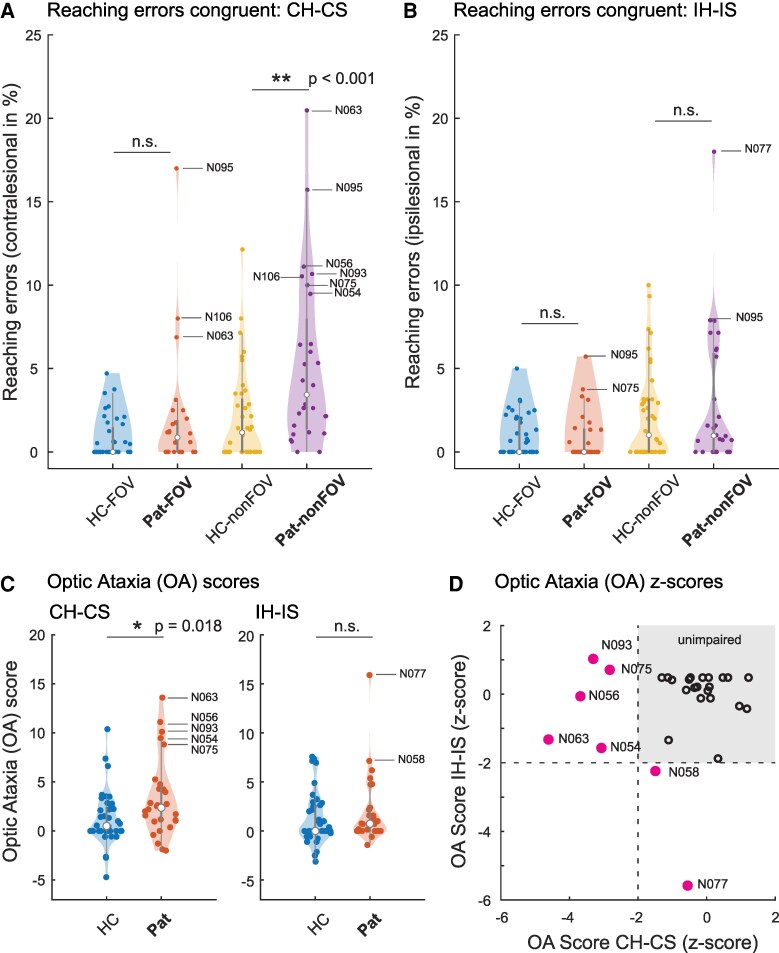
**Reaching errors and OA scores as a function of hand and space and viewing conditions.** Congruent condition refers to the same hand and space (*n*_patients_ = 28; *n*_healthy_ = 40). All tests between thalamic patients (Pat) and HCs were carried out as Mann–Whitney U-tests (two-sided) as a function of hand and viewing condition. In **A** and **B**, ‘FOV’ refers to the foveal condition, where subjects were allowed to look at the pen, while ‘nonFOV’ refers to the non-foveal (peripheral) condition, where subjects fixated on the camera. (**A**) Reaching errors for the contralesional hand and space (CH–CS), comparing foveal and non-foveal conditions. When subjects were allowed to fixate on the objects (‘FOV’), reaching errors in the healthy subjects did not significantly differ from the thalamic patients (*U* = 683.0, *P* = 0.09) in the CH–CS. When subjects were not allowed to look at the objects (‘nonFOV’), patients made significantly more reaching errors (*U* = 820.0, *P* = 0.001). **The original *P*-value, significant after Bonferroni correction (*α* = 0.00125) (**B**) Reaching errors to the ipsilesional hand and space (IH–IS). No significant difference was found between patients and controls in the foveal (*U* = 521.0, *P* = 0.59) nor in the non-foveal viewing condition (*U* = 602.0, *P* = 0.59). Thus the patient group showed the largest numbers of errors for peripheral reaches with the contralesional hand and contralesional space. (**C**) Distribution of OA scores in patients and HCs as a function of hand and viewing condition. OA scores represent the difference of reach errors in the peripheral (non-foveal) condition minus reach errors in the foveal condition, calculated separately for each hand/space combination. Note the shifted median scores for the CH–CS condition and the few patients with higher scores than the main group (HC versus patients in the CH–CS condition: *U* = 749.5, *P* = 0.018; *the original *P*-value, significant after Bonferroni correction (two conditions, *α*-level = 0.025) HC versus patients in the IH–IS condition: *U* = 639.0, *P* = 0.319). In the violin plots, the white dots represent the median, the thick grey bar the interquartile range, the thin grey lines (‘whiskers’) represent 1.5 times the IQR above the third quartile and below the first quartile. (**D**) Scatter plots showing the *z*-scored contra- and ipsilesional OA scores in the patient group. As in neuropsychological convention, the sign of the *z*-scores was flipped so that negative values represent worse performance. The labeled lines represent the patients with an OA *z*-score lower than two SD from the HCs (also significant in the Crawford–Garthwaite test for single subjects).^35^

We examined 28 patients with circumscribed thalamic lesions. Participants were asked to reach and grasp a pen presented at different peripheral locations (Fig. 1). To test for OA, trials that required eye fixation on the camera in front of the patients, and trials in which patients were asked to look at the pen, were interleaved. Reach performance in each patient was assessed by independent raters scoring the videos for reach smoothness and precision. The reach results are shown in Fig. 3.

### Reaching errors in foveal and peripheral conditions

We first compared the reach performance between patients and HCs. In the OA literature, reach errors are typically distinguished by hand, space and viewing condition.^3,4^ From the video ratings, an individual percentage error score was calculated for each of the hand, space and viewing conditions (Materials and methods). For simplicity, we focus the main figures on the OA-relevant congruent hand–field conditions, where hand and side of the pen match.^3^ The mixed repeated-measures ANOVA with the between-subjects factor *GROUP* (healthy subjects versus patients) and the two within-subjects factors: *VIEWING CONDITION* (foveal versus peripheral) and *SIDE* (CH_CS versus IH–IS) revealed a main effect of *GROUP* [*F*(1,66) = 6.0, *P* = 0.017, *η*p^2^ = 0.083]. The interaction between *GROUP* × *SIDE* was also significant [*F*(1,66) = 7.68, *P* = 0.007; *η*p^2^ = 0.10]. The critical interaction for the definition of an OA, the interaction between GROUP × *VIEWING CONDITION* was also significant [*F*(1,66) = 5.88, *P* = 0.018; *η*p^2^ = 0.082]. The three-way interaction between GROUP × SIDE × *VIEWING CONDITION* did not reach significance [*F*(1,66) = 1.63, *P* = 0.21; *η*p^2^ = 0.024]. In the two sections below, we present the data in detail, including the respective non-parametric *post hoc* tests. Medians and ranges for all groups, including the not so relevant crossed (incongruent) conditions are presented in Supplementary Table 3.

### Reaching errors: foveal condition

Apart from a few exceptions, healthy subjects rarely made any reach errors when they were allowed to look at the pen, yielding median error scores of 0 [assigned ‘contralesional’ hand/space combination (CH–CS): range 0–4.7%; assigned ‘ipsilesional’ hand/space combination (IH–IS): 0–5.0%] (Fig. 3A; Supplementary Table 4). Similarly, only few thalamic patients made errors in the foveal condition, either in the contralesional hand/space (CH–CS) (median: 0.9%, range: 0–17%) or ipsilesional hand/space combination (IH–IS) (median: 0.9%, range: 0–5.7%). Accordingly, the Mann–Whitney U-test did not yield a significant result for the comparison between patients and the HC group in the foveal condition (CH–CS: *U* = 683.0, *P* = 0.099; IH–IS: *U* = 521.0, *P* = 0.593). In the foveal condition, the U-test also lacked significance for the incongruent conditions (CH-IS: *U* = 618, *P* = 0.422; IH-CS: *U* = 521, *P* = 0.478). Separating the percentage of corrected and uncorrected errors yielded a similar picture. Very few errors occurred in the foveal condition for either hand/space combination.

### Reaching errors: non-foveal (peripheral) condition

From the (cortical) OA literature, we expected the highest error rates for reaches with the contralesional hand to the contralesional space and for the peripheral viewing condition.^2,7^ Since reach–grasp movements are more difficult to execute when we do not look at the objects, healthy subjects and patients had higher error rates in this peripheral (non-foveal) condition (Fig. 3A and B; Supplementary Table 4). Critically, as a group, patients made significantly more errors than the HCs when using their contralesional hand towards the contralesional space in this peripheral condition [median: 3.4%, range: 0–20.5%; *U* = 820.0, *P* < 0.001 (passing the Bonferroni-corrected *α* = 0.0125); Fig. 3A]. None of the other hand–field combinations yielded a significant group effect (all *P* > 0.15). In terms of percentage of corrected errors in the non-foveal CH–CS condition, the median of the patients was 10.0% with a large range from 0 to 69%. Uncorrected errors were rare, resulting in an overall median of 0 (range: 0–7.1%) even in the CH–CS condition. In the ipsilesional hand/space condition, there were less corrected and uncorrected errors overall, yielding a median of 3.7% (range: 0–39.3%) of corrected and 0% uncorrected errors (range: 0–13.3%) (Supplementary Table 4).

### OA scores

The higher amount of reach errors in the contralesional peripheral condition already hinted at the occurrence of OA in the thalamic patients. The defining feature of OA is the disproportionate occurrence of errors when objects are in peripheral space, i.e. are not foveated.^2^ To test directly for OA, we next subtracted the individual error scores in the foveal condition from the error scores in the peripheral condition.^3^ As a group, thalamic patients had significantly higher OA scores than HCs when using their contralesional hand to reach into the contralesional space (*U* = 749.5, *P* = 0.018) (Fig. 3C). It is also clear from this figure that the significance at the group level is driven by five individual patients with high OA scores. Arguably, those patients are more important than the group mean and will be detailed below. On the group level, there was no significant deficit when patients used their ipsilesional hand and reached to the ipsilesional space (OA score IH–IS, *U* = 639.0, *P* = 0.32). Figure 3D shows the *z*-score transformed regression plot between the OA scores for the combination CH–CS and IH–IS conditions. A negative *z*-score indicates higher OA scores, and a threshold of −2 indicates a performance worse than 2 SD from the HCs. Five thalamic patients had a poorer performance in the CH–CS condition, and two patients had a poorer performance in the ipsilesional (IH-IS) condition (Fig. 3D). The median and ranges for all hand/eye conditions are presented in Supplementary Table 4.

### Selective case analysis

**Figure 4:**
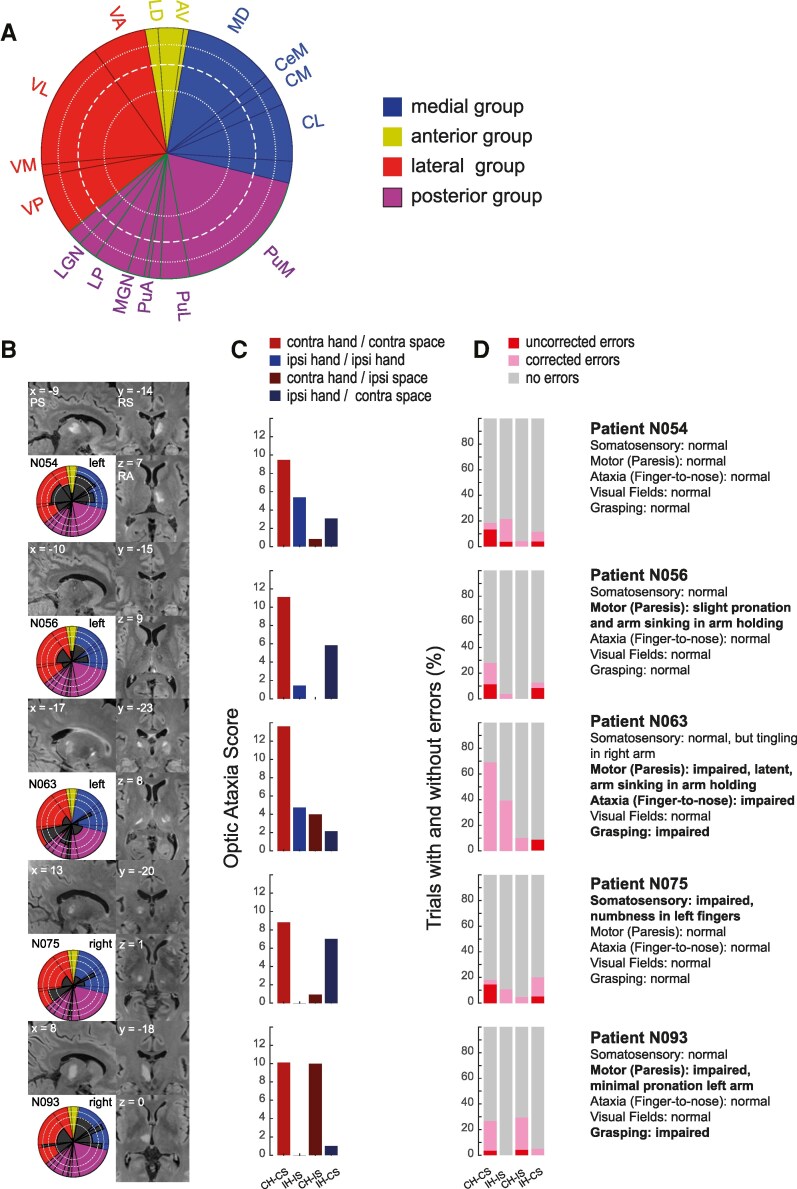
**Selective case analysis: patients with pathological OA scores in the CH/CS condition (*n*_patients_ = 5).** (**A**) Morel Atlas scheme. AV, anterior ventral nucleus; CL, central lateral nucleus; CeM, central medial nucleus; CM, centromedian nucleus; LD, lateral dorsal nucleus; LGN, lateral geniculate nucleus; LP, lateral posterior nucleus; MD, mediodorsal nucleus; MGN, medial geniculate nucleus; PuA, anterior pulvinar; PuI, inferior pulvinar; PuL, lateral pulvinar; PuM, medial pulvinar; VA, ventral anterior nucleus; VL, ventral lateral nucleus; VM, ventral medial nucleus; VP, ventral posterior nucleus. (**B**) Individual anatomical scans (FLAIR) were registered to the high-resolution MNI 152 template to visualize the thalamic lesions. Schema shows the distribution of the lesion on the morel scheme. Black areas in the Morel Atlas scheme indicate the amount of damage to thalamic substructures. White dotted and dashed circles indicate 25, 50 and 75% damage. RS, right superior; RA, right anterior; PS, posterior superior, *x*, *y*, *z* are the MNI coordinates, the number on top of each figure is the patient’s acronym. (**C**) OA scores for each patient (non-foveal minus foveal errors), separated by the hand and space. (**D**) Percentage corrected and uncorrected errors in the non-foveal condition. The text depicts the main clinical features of the patient. CH–CS, contralesional hand-contralesional space; IH–IS, ipsilesional hand-ipsilesional space; CH–IS, contralesional hand-ipsilesional space; IH-CS:,ipsilesional hand-contralesional space. The number of valid trials for each patient were as follows (Foveal/Non-Foveal): N054: CH–CS: 17/19, IH–IS:17/14, CH_IS: 13/12, IH_CS: 13/14; N056: CH–CS: 15/18, IH–IS: 20/15, CH_IS: 13/13, IH_CS: 15/13; N063: CH–CS: 16/21, IH–IS: 16/14, CH_IS: 14/10, IH_CS: 14/12; N075: CH–CS: 17/14, IH–IS: 16/19, CH_IS: 15/13, IH_CS: 15/10; N093: CH–CS: 20/15, IH–IS: 15/20, CH_IS: 13/12, IH_CS: 13/10.

Since OA studies in thalamic patients are exceedingly rare, we here present some details of the five individual OA cases to show the range of space and/or hand-specific effects and their putative relationship to the thalamic lesion sites (Fig. 4). Figure 4A and B depicts the MR-based lesion centres of each patient, with a descriptive thalamic nuclei scheme next to it. Exact lesion locations for each patient are shown in Supplementary Table 1. Figure 4C and D depicts the OA scores and the proportion of corrected/uncorrected errors of each patient, respectively. The combination of contralesional hand/space (CH/CS) was typically the worst condition, while the hand and space could be affected to varying amounts. In the group of patients with contralesional OA, the majority of patients made mostly corrected errors (Fig. 4D). Somatosensory impairments, including misperceptions in the upper limbs, were not a prerequisite for OA. Furthermore, an abnormal performance in the arm holding test (mild paresis) was often documented in the records, while this modest sinking of the arm with closed eyes could also be attributed to proprioceptive deficits. For example, patient N063, with the most pronounced deficits, had almost symmetrical bilateral lesions in the medial (CM, CL) and posterior portion (Pulvinar, LP), while the lateral lesions (VL, VM, VP) were more pronounced in the left hemisphere. In the standard clinical testing, the right lesion remained clinically silent, such as tingling of the arm and dysmetria in the FNT, which occurred on the right side only (see Patient Descriptions in the Supplementary material). In the OA task, N063 made a large amount of corrected errors in the non-foveal condition with the right hand. In those trials, only 30.9% trials with this hand were rated effective and fluent, while the remaining trials were slowed and ineffective but were corrected in flight. The slowed and corrected reaches with the right hand in the right space led to a pathological OA score in this patient (*z* = −4.6). The ipsi hand/field OA condition was not impaired (OA *z*-score: −1.32) (Fig. 4, middle panel). She also made uncorrected errors, while those are likely underestimated in our cohort, as patients sometimes looked at the pen when they did not reach it, in which case the trial was invalidated. Grasping data of the whole population are shown in Supplementary Fig. 3 and Table 5. Only two of the five OA patients showed grasp impairments when they were allowed to look at the small items. Thus, on the thalamic level, OA and grasping impairments can dissociate. The extended case studies of each shown patient can be found in the Supplementary material (Single Patient Descriptions, Supplementary Table 3). The two patients with ipsilesional deficits showed a pattern that is hard to interpret: the first patient (N058) had only a minor deficit for both the CH–CS and the IH–IS conditions (CH–CS: *z* = −1.4; IH–IS: −2.24). Patient N077 (no neglect, VEP’s intact) had a strong ipsilesional space effect, exhibiting the strongest deficits for the IH_IS and CH_IS conditions (*z* = −5.58 and *z* = −9.74). More detailed demographic, clinical and neuropsychological information of each patient can be found in Supplementary Table 3.

### Patients with and without OA

We next focused on the question, which clinical variables discriminate between thalamic patients with and without OA on the population level? To this end, we used the *z*-transformed OA scores based on the HC group. We then compared the patients who had an OA score worse than 2 SD with the patients with normal *z*-scores (Fig. 3C and D). The group of patients with contralesional OA versus without contralesional OA (*n* = 5) did not differ in age, sex, aetiology nor in their basic clinical parameters such as somatosensory, motor or grasping deficits (Mann–Whitney or *χ*^2^-test, where appropriate, Table 2). Similarly, none of the comparisons reached significance when adding the two OA patients with ipsilesional OA and comparing the resulting OA group (*n* = 7) with the non-OA patients (*n* = 21) (all *P* > 0.1). OA occurred after left or right thalamic lesions (Table 1), whether there is some laterality needs to be addressed with larger sample sizes. One contributing factor was whether the thalamic lesion was unilateral or bilateral. In the group of contralesional OA patients, three out of five (60%) had also a smaller lesion on the other side (Fig. 4B). In contrast, only 3 out of 23 (13%) patients without OA had a bilateral lesion (*χ*^2^ = 5.38, *P* = 0.02). Since all patients were investigated in the days following their stroke during their stay in the clinic, not all patients received cognitive testing or detailed neuropsychological testing. Nonetheless, MMSE or MoCA were conducted in 19 patients, but only in 2 out of the 5 OA patients. Patients without OA achieved a mean of 27.94 points (range: 22–30), and the OA patients achieved a mean of 27.00 (range: 24–30). Since the OA patient with the highest OA error scores (N063) achieved the full score, OA occurs even in patients without apparent cognitive deficits.

**Table 2:** Demographic and clinical data of all patients who presented with and without pathological OA scores

	Statistical measure	OA patients	Non-OA patients	Statistical test	*P*-values
Number of patients	*N*	5	23		
Handedness	%per cent right	100	96%	Chi-square (*χ*^2^)	0.635
Age	Years	55.8 [24.8, 71.2]	59.6 [32.3, 80.5]	Mann–Whitney *U*	0.88
Sex	*N* Sex males	4	17	Chi-square (*χ*^2^)	0.776
Aetiology	*N* Infarct	4	22	Chi-square (*χ*^2^)	0.331
Lesion side	Lesion side (left)	3	11	Chi-square (*χ*^2^)	0.866
Bilateral lesion	*N* present (%)	3 out of 5 (60%)	3 out of 23 (13%)	Chi-square (*χ*^2^)	0.02^a^
Lesion size	mm^3^	1802.0 [781.3, 2085.8]	1089.1 [170.8, 5712.9]	Mann–Whitney *U*	0.139
Time since lesion	Median days	6.0 [3.0, 9.0]	4.0 [2.0, 9.0]	Mann–Whitney *U*	0.792
Somatosensory upper contra	%present	20	43.5	Chi-square (*χ*^2^)	0.33
Somatosensory lower contra	%present	0	30.4	Chi-square (*χ*^2^)	0.154
Paresis upper contra	%present	60	47.8	Chi-square (*χ*^2^)	0.622
Paresis lower contra	%present	20	34.8	Chi-square (*χ*^2^)	0.521
Ataxia contra	%present	20	13.0	Chi-square (*χ*^2^)	0.687
Aphasia	%present	20	8.7	Chi-square (*χ*^2^)	0.459
Neglect	%present	0	8.7	Chi-square (*χ*^2^)	0.635

^a^The significant *P*-values.

### Lesion locations in patients with and without OA

**Figure 5:**
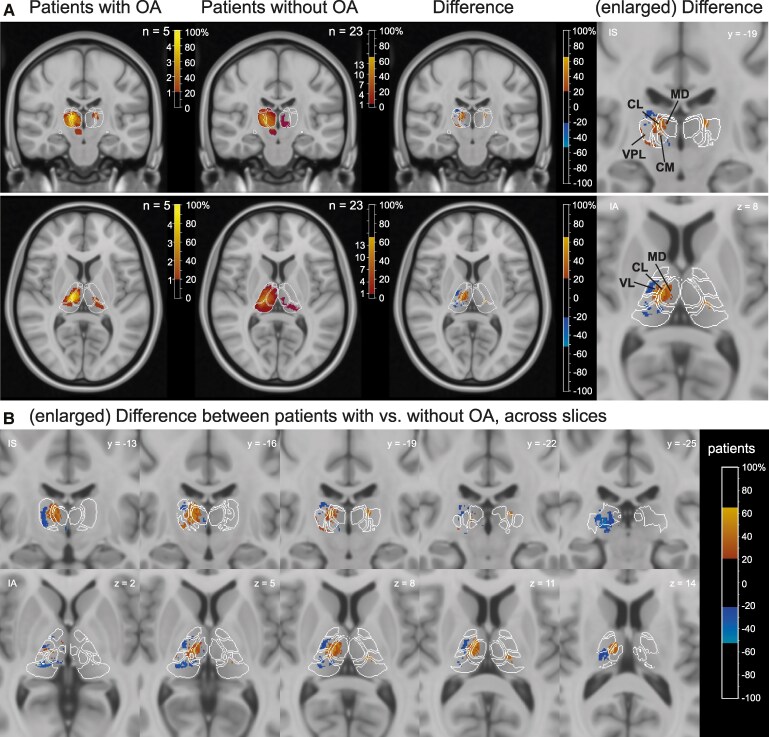
**Lesion overlap and lesion subtraction plots of the patients with and without OA.** (**A**) Lesion overlap and lesion subtraction of the patients with (*n*_patients_ = 5) and without OA (*n*_patients_ = 23) overlaid onto the MNI template in a coronal (upper row, *y* = −19) and an axial view (lower row, *z* = 8). Primary left thalamic lesions were flipped to the right hemisphere (see Supplementary Fig. 4 for non-flipped maps). Shown are the lesion overlaps of impaired (first column, *n* = 5) and unimpaired patients (second column, *n* = 23) and the lesion subtraction, i.e. the group difference (third column), which was calculated by subtracting the percentage lesioned in the group without OA from the percentage lesioned in the group with OA. The heatmap shows the number of patients (*n*) on the left and the percentage of patients on the right (%). The rightmost column shows the enlarged subtraction plots of these slices. (**B**) Systematic views of the lesion subtraction for OA across coronal and axial thalamic slices. Positive values in lesion subtraction plots in **A** and **B** in red-yellow indicate stronger lesion load in patients with OA, whereas negative values in blue indicate stronger lesion load in patients without OA. For visualization purposes, group differences are displayed when they exceeded 20%. White outlines represent the borders of selected thalamic nuclei as defined in the digital version of the Morel-atlas normalized to the 0.5 mm MNI152 T1 template. VL, ventral lateral posterior nucleus; VPL, ventral posterior lateral nucleus; MD, mediodorsal nucleus; CL, central lateral nucleus; CM, centromedian nucleus. Thalamic nuclei on selected coronal and axial slices are depicted in Supplementary Fig. 5. Lesion locations for each patient and summary percentages are shown in Supplementary Tables 1 and 2. In **A** and **B**, the numbers refer to the MNI coordinates of the *y*-axis (coronal slices) and *z*-axis (axial slices), respectively. I, ipsilesional for flipped; A, anterior; S, superior.

In order to identify the critical lesion sites for patients with and without OA, we next contrasted the lesions of the five patients with (contralesional) OA to thalamus lesions in patients without OA. Figure 5A shows the most common lesion sites in the thalamic patients with and without OA. Based on the (cortical) literature, we hypothesized that OA should occur in patients with lesions in thalamic nuclei with strong connectivity to SPL/IPL—i.e. VPL, VL and LP/pulvinar. Since we did not find a hemispheric trend for OA scores (Mann–Whitney U-test, *P* = 0.87, Table 2), we flipped all lesions to the right hemisphere. In the group of the five OA patients, 100% had a lesion that involved the lateral thalamus and in 80% (*n* = 4) also the medial thalamus (Fig. 5A, left panel). The posterior group was only involved in one OA patient. From the medial group, MD was damaged in 40% (MDmc in 40% and MDpc in 60%). Within the medial group, where the ILN are placed according to the Morel atlas, CL and CM were affected the most frequently (80%). From the lateral group, VPL was affected in 100% and VL in 80%. Within the posterior group, pulvinar or LP was damaged in only one patient. In order to identify the thalamic nuclei that are typically affected in patients with OA, it is important to directly contrast the patients with and without OA. Given the differing vasculature-induced differences in the likelihood of stroke affection, the relative percentages between lesions in OA versus non-OA lesion controls are important. For example, a CL lesion was present in 80% of the OA patients, but only in 43.4% of the patients without OA (Fig. 5A, middle panel). Similarly, a VL lesion was present in 80% of the OA patients but only in 47.8% of the patients without OA. VPL was affected in all OA patients, but only in 52% of the non-OA patients. In pulvinar and LP, the pattern is reversed: only 20% of OA patients had pulvinar or LP lesions, but 43.4% of patients without OA had a lesion there (Fig. 5A, middle panel). Thus, to identify the thalamic nuclei that are commonly lesioned in OA patients but spared in the OA, we subtracted the lesion overlays of patients without OA from the OA patients (Fig. 5A, right panel, Fig. 5B). The non-flipped data on the original hemisphere are shown in Supplementary Fig. 4. The thalamic structures that were commonly damaged in patients with OA but were typically spared in patients without OA were the lateral MD, CL, CM, VPL and VL, with a concentration at the border between them, i.e. the ILM. Pulvinar nuclei did not belong to this group of lesions that were commonly damaged in OA patients but were typically spared in patients without OA. Schematic borders of the thalamic nuclei can be found in Supplementary Fig. 5.

### Control analyses

Although the lesion subtraction method should remove unspecific effects that are due to the general lesion distribution, we conducted several control analyses to address the specificity in our sample. To this end, we computed the lesion subtraction for patients with and without paresis of the upper limb (*n* = 14 versus *n* = 14). Supplementary Figure 6A and B shows that VL and VPL were the most commonly affected in patients with contralesional limb problems. In contrast, lesions of patients with and without contralesional somatosensory deficits (including paraesthesia, pain, etc.; *n* = 11 versus *n* = 17) centred on VPL and the anterior pulvinar. Similarly, when we computed the lesion subtraction to compare patients with and without deficits in a fine motor grasping task (*n* = 8 versus *n* = 20), sites that were commonly affected in patients with grasping difficulties but were typically spared in patients without also centred on VPL and anterior pulvinar (Supplementary Methods and Fig. 7). This suggests that grasping deficits may be due to the somatosensory and/or sense of position impairments in those patients. Nucleus delineations for all panels are available in Supplementary Fig. 5. Thus, in contrast to OA, other deficits such as motor, somatosensory or grasping symptoms affected different thalamic regions and did not centre on the border between lateral MD and VL. All difference maps were distinct from the difference we found relevant for OA.

## Discussion

Our analysis of 28 stroke patients revealed that OA can occur as a result from circumscribed thalamic lesions. In fact, in 5 of these 28 thalamic patients, we found typical OA behaviour. Reaches with the contralesional hand towards the contralesional space were particularly impaired, similar to a number of previous studies in OA patients with cortical lesions.^1,7^ This combination of hand and field effects in (unilateral cortical lesion-induced) OA has been taken as evidence that OA is caused by a problem to transform visual input into motor output and/or between spatial coordinate frames.^6,36^ OA in our thalamic patients could not be explained by either primary sensory (visual or somatosensory), motor deficits or spatial neglect. The direction of misreaches was not necessarily towards the central fixation, thus we did not observe systematic ‘magnetic misreaches’.^37-39^ Although grasping deficits and OA occurred frequently together, they did not always co-occur, similar to some OA patients with cortical lesions.^40^ The lesion subtraction analysis of 28 thalamic stroke patients with and without OA showed a region in both hemispheres that centred around the ILM and included parts of CL, lateral MD (i.e. MDpc), VPL and the medial portion of VL. Pulvinar lesions, despite its strong connectivity with the parietal cortex, were surprisingly not associated with OA. The lesion pattern associated with OA was clearly distinct from the lesion patterns that led to paresis, somatosensory or grasping deficits with small items in the centre of gaze. Instead, lesion sites associated with grasping deficits in foveal vision and with small items, primarily involved VPL and the anterior pulvinar. The grasping deficits following pulvinar lesions are consistent with both human^23^ and monkey lesion studies,^24,25^ and it is possible that subportions such as the anterior pulvinar portion are particularly important for this to occur.

### The central thalamus as the critical lesion site for OA

The critical lesion site we found in OA patients was not restricted to one circumscribed region within the Morel atlas system. Instead, it encompassed several nuclei within and close to the internal medullary laminar (IML) complex. Interestingly, this region matches the so-called ‘central thalamus’, also sometimes called ‘oculomotor thalamus’. Since the pioneering electrophysiological studies in awake monkeys, it is considered to include the anterior group of the ILN (CL, CM), but also adjacent regions such as the medial portion of ventrolateral (VL) and the lateral portion of the mediodorsal thalamus (MD).^41-43^ This ‘central thalamus’ region matches our OA-relevant thalamic lesion location quite well, while specifics can be recovered for each of the subregions.

CL and CM are part of the ILN that are embedded within the IML, a fibre mass that separates the medial from lateral thalamus nuclei.^44-46^ CL/CM project to widespread cortical areas, including the frontal cortex but also basal ganglia, playing a role in modulating consciousness and cortical activity. This has been demonstrated by arousal- and consciousness-enhancing effects of microstimulation in anaesthetized non-human primates,^47^ which were accompanied by increased fMRI BOLD activity in prefrontal, parietal and cingulate cortices.^48^ Similar effects of microstimulation in the central thalamus were reported in patients with severe brain injuries.^49^ Furthermore, previous case reports in human stroke patients report memory problems following small IML lesions that are likely due to frontal-type executive deficits.^50-52^ In all cases, those cognitive deficits following IML lesions were interpreted as consequences of a functional connectivity breakdown across long-range cortico-cortical pathways and within cortico–striatopallidal–thalamocortical loop connections.^49,50^ We follow this interpretation and suggest that OA following lesions in CL/CM might be explained by similar activity breakdowns, likely in fronto-parietal circuits, which needs to be investigated in further electrophysiological and fMRI studies. The VL complex is part of the classical ‘motor thalamus’, known to play a central role in motor control by processing and conveying information from the basal ganglia and cerebellum to motor cortex.^13,14^ This integration is essential for the fine motor coordination, planning and execution of voluntary movements, as shown in previous human patient studies.^53-56^ Finally, similar to the nuclei complexes mentioned above, the mediodorsal thalamus (MD) also contains several subportions, and its lateral portion (sometimes called MDmf or MDpc) that is reciprocally connected with lateral frontal cortical regions (dorsolateral/dorsomedial prefrontal cortex) is critical for cognitive control, working memory and executive functions.^57^ This lateral MD portion has also been studied in the context of eye movements, while reach–grasp studies are lacking.^13^ In an elegant study that tested the hypothesis that this MD portion carries corollary discharge signals from superior colliculus to FEF, Sommer *et al*. pharmacologically inactivated this region while monkeys performed double-step saccades. They provided evidence that the lateral MD conveys signals about outgoing motor commands to frontal cortices.^58,59^ While this is speculative in the context of our study due to the absence of concurrent electrophysiological or functional imaging measures, a potential link between a failure of corollary discharge and OA could lie in the disruption of internal predictive mechanisms that normally update spatial representations during movements.^59^ Since visual information is less reliable in the periphery, i.e. when the target cannot be fixated, the movement planning may need to rely more on internal (e.g. proprioceptive) and corollary discharge information. This, in turn, might be impaired with lesions in these central thalamus regions that contain CL, CM, VL and the lateral MD. Given that OA deficits were mostly side and hand-specific, and instructions were given before each trial, this cannot be explained by a general breakdown of executive or memory functions.

Apart from the functional properties of central thalamus neurons mentioned, they often also exhibit persistent activity as a function of eye position,^60,61^ as in many sensorimotor cortical regions such as parietal cortex.^62-65^ Strong support for the importance of the central thalamus to eye position perception is further provided by lesion studies in humans, showing impairments in perceiving and integrating eye position information across saccades.^66-68^ Eye position information is thought to be critical for transformations between retinal and egocentric limb/body coordinates that are relevant for visually guided reaches.^69^ OA following lesions in this thalamic nucleus complex could then be construed as a failure to transmit correct eye position information to parietal cortex, which then impairs the integration of retinal, eye and hand signals as a prerequisite of reaching for visual objects. Or not mutually exclusive, as a breakdown of parietal cortex capacity to integrate different directional eye- and hand-related information, thus depriving motor-related areas in prefrontal cortex of important visuomotor inputs.^70^

### Lack of OA following pulvinar lesions

To our great surprise, patients with lesions in the pulvinar did not typically exhibit OA. Based on its reciprocal connectivity to fronto-parietal regions that subserve visuomotor functions^71-73^ and importantly to the OA-linked SPL/IPL regions,^8^ we had hypothesized that (dorsal) pulvinar lesions would preferentially lead to OA. Furthermore, there were two pulvinar inactivation/lesion studies in macaque and marmoset monkeys who reported ‘OA’ following pulvinar lesions.^24,25^ The first study in macaques inactivated the dorsolateral portion of the pulvinar and reported imprecise and slowed reach-grasping along with failures to appropriately preshape the hand, most pronounced with the contralesional hand and space.^25^ Another study in marmosets showed that ablation of the ventral pulvinar in early life results in persistent misreaches and grasping deficits.^24^ Similar to the ‘central thalamus’ detailed above, neurons in ventral and dorsal pulvinar portions are strongly modulated by eye movements^74,75^ and many pulvinar neurons carry information about (static) eye position during fixation and code visual stimuli in a dynamic spatial reference frame, from predominantly eye-centred visual encoding to final gaze representations in non-retinocentric (possibly body-centred) coordinates.^76^ The few studies, which recorded pulvinar neurons in reach tasks in monkeys, reported neurons that either responded during reach planning, execution or post-reach.^77-79^ On the other hand, some basic reach properties of pulvinar neurons such as specificity for hand (left, right) and hand/space interactions and the underlying spatial reference frames remain unknown.^80^ It is not clear why damage in other higher-order thalamic nuclei (i.e. central thalamus) with similar connectivity should be more likely to induce OA. Since we excluded patients with visual field loss and thus LGN lesions, we have likely excluded patients with damage of the posterior choroidal artery that also supplies the more ventral portion of the pulvinar.^55^ Hence, our sample was biased towards more medial/anterior pulvinar portions. Further lesion studies should also selectively include more dorsolateral pulvinar portions that have strong parietal connectivity as well.^81,82^

### Limitations

Ischaemic lesions are typically not restricted to a single nucleus but occur in combinations defined by the vascular supply.^55^ The anatomical specificity is thus somewhat limited by the stroke aetiology in our patients, although the lesion subtraction method should largely correct for this.^83^ Unfortunately, the number of OA patients was too small to allow for statistically rigid voxel-based symptom-lesion mapping (VLBM).^84,85^ Thus, future studies need to increase the number of thalamic stroke patients. Digital movement tracking with extraction of time courses would also have helped to quantify more subtle changes of reach–grasp behaviour, such as slowing. Since we did not systematically vary head and trunk position, we cannot say whether our patients had a specific problem with eye-based reference frame transformations or whether they had problems in other, i.e. head-based or trunk-based spatial reference frames.^63^ While we conducted computer-based visual tasks to control for general vision and spatial attention problems, we do not have a systematic assessment of proprioceptive deficits. Future studies should also test whether the misreaches are specific for the visual modality or whether they would occur when pointing towards auditory or somatosensory targets.^86^ Furthermore, based on the time/medical care constraints in our hospitalized stroke patients, we only conducted short cognition tests in some patients (MoCA/MMSE). Future studies might conduct thorough neuropsychological assessments. We do not think that, e.g. memory or cognitive deficits interfered with performance in this study, as the tasks applied were also employed in monkeys before, and task instructions were given and monitored in each trial by the experimenter. Since the critical thalamic lesion site for OA is distributed through several thalamic nuclei as defined by structural anatomy, a lesion-based network mapping (LSM) or resting-state fMRI approach could be useful in future studies to understand the remote network effects better.^87-90^

## Conclusion

We analysed 28 stroke patients with circumscribed thalamic lesions and detected five patients who resembled the well-known OA as observed in patients with parietal lesions. The critical lesion site appears to be a group of nuclei often referred to as ‘central thalamus’.

## Supplementary Material

fcaf359_Supplementary_Data

## Data Availability

Anonymized data may be shared on request to the corresponding author from a qualified investigator for non-commercial use, subject to restrictions according to participant consent and data protection legislation. Lesion masks and MATLAB code for the lesion analysis is provided in https://github.com/MelanieWilke/Optic-Ataxia.
