# Supplementary material for: Optic ataxia in patients with thalamic lesions
Source: Brain Commun. 2025 Sep 13;7(5):fcaf359. doi: 10.1093/braincomms/fcaf359 (PMC12484446; doi:10.1093/braincomms/fcaf359)
Supplement: fcaf359_Supplementary_Data [file fcaf359_supplementary_data.pdf]

Supplementary material

Optic Ataxia in Patients with Thalamic Lesions

Melanie Wilke[1,2,*,#], Hanna J Eisenberg[3,#], Carsten Schmidt-Samoa[1,#], Shirin Mahdavi[1], Igor Kagan[1,4], Peter Dechent[1], Jan Liman[3,6], Hans-Otto-Karnath[5], Mathias Bähr[3]

[1] Department of Cognitive Neurology, Heart & Brain Center, University Medical Center Goettingen, Goettingen, 37075, Germany

[2] Cognitive Neurology Group, Cognitive Neuroscience Laboratory, German Primate Center – Leibniz Institute for Primate Research, 37075, Goettingen, Germany

[3] Clinic of Neurology, University Medical Center Goettingen, Goettingen, 37075, Germany

[4] Decision and Awareness Group, Cognitive Neuroscience Laboratory, German Primate Center – Leibniz Institute for Primate Research, 37075, Goettingen, Germany

[5] Center of Neurology, Division of Neuropsychology, Hertie-Institute for Clinical Brain Research, 72076, University of Tübingen, Tübingen, Germany

[6] Department of Neurology, University Medicine Center Nuremberg, Paracelsus Private University, 90471 Nuremberg, Germany

# Melanie Wilke, Hanna Eisenberg and Carsten Schmidt-Samoa contributed equally

Supplementary material includes:

Supplementary Methods

Single Patient Descriptions

7 Supplementary Figures

5 Supplementary Tables

Supplementary Methods

Testing for spatial neglect

Testing for spatial neglect was conducted with several paper and pencil tests of the German neglect battery [1]: (i) line bisection, (ii) line cancellation, (iii) star cancellation and the Apples test [2]. All these paper-based tests were presented on a horizontally-oriented DIN A4 sheet. Time of test completion depended on the patient indicating to be satisfied with his/her performance. In order to assess the spatial bias we calculated the center of cancellation (CoC) with custom-written software similarly to [3]. In short, the CoC is a continuous measure that represents the mean horizontal coordinate of the detected items, first in terms of centimeters and then transformed into a normalized score [4], based on the performance of our group of healthy controls. Apart from the paper and pencil tasks, visual attention deficits were tested with the Posner paradigm, shown to be sensitive even for patients with mild neglect [5,6]. The task design and temporal structure of the Posner task was adapted from previous neglect studies [7]. In the computerized paradigm which we adopted from [7], subjects detect a visual stimulus presented to the left or right from fixation. The side of attention is cued by an additional stimulus that appears before the target appearance. Critically, in the majority of trials (75%, 'valid'), the spatial cue correctly predicts the side of target appearance, while pointing towards the opposite hemifield in the remaining trials (typically 25%, 'invalid'). Three key parameters were derived from the Posner-Task: i) Longer detection latencies (and/or a higher number of misses) for contralesional items indicate deficits in visual perception/attention ('side/visual field effect'); ii) Larger response delays between trials with valid cues vs. invalidly cued location indicate deficits in re-orienting attention ('validity effect'); iii) Unproportionally longer response delays for contralesional targets when the ipsilesional side was cued (i.e. most likely attended) are taken as deficits to disengage attention ('disengagement effect'). For our neglect assessment we used the reaction times for contralesional targets. A z-score smaller than -2 in more than 2 of the 5 tasks (paper and pencil tests and Posner task) was considered as spatial neglect.

Assessment of grasping performance

We assessed fine visually-guided reaching-grasping abilities of the patients and the control group by letting them pick small objects ('Smarties' candies: oblate spheroids with a minor

axis of about 5 mm and a major axis of about 12 mm) from the center and peripheral table positions. The task and scoring was adopted to be comparable to our previous pulvinar lesion studies in non-human primates and humans [8,9] (**Supplementary Fig. 1**). Participants were always allowed to look at the objects. We calculated an error score (fluent (0), slowed (1), corrected during (2) / after the first (3) or the second (4) attempt, not successful (5). Subjects performed 36 (median) reach-grasp trials per session with either the left or right hand. Healthy subjects rarely made any reach-grasp errors while grasping the objects irrespective of hand or spatial position, yielding a median error score of 0. Since there were more than two conditions, for the grasping scores we calculated a mixed repeated measures ANOVA with the between-subjects factor *GROUP* (healthy subjects vs. patients) and two within-subject factors: *Hand* (contralesional vs. ipsilesional) and *Space* (contralesional vs. ipsilesional). Since the grasping scores compared the two groups in four conditions, we assumed $\alpha = 0.05$ as original and a corrected α ($0.05/4 = 0.0125$) for the post-hoc comparisons. In the description of the ANOVA results we focus on the relevant *GROUP* and their interaction effects, i.e. *GROUP x HAND*, *GROUP x SPACE* and *GROUP x HAND x SPACE* effects. There was a significant main effect of *GROUP* ($F(1,66) = 14.163$, $p < 0.001$; $\eta p^2 = 0.177$), indicating that the patients had an overall grasping impairment. There was also a significant interaction between *GROUP* x *HAND* ($F(1,66) = 5.183$, $p = 0.026$, $\eta p^2 = 0.073$). As can be appreciated from **Supplementary Figure 3**, patients with thalamic lesions exhibited stronger reach-grasp impairments for the contralesional hand. Consequently, the Mann-Whitney U test for the group comparison contralesional hand into the contralesional space reached significance (CH-CS: U=273.5, p<0.001) as well as the group comparison for reach-grasp movements with the contralesional hand into the ipsilesional space (CH-IS: Mann-Whitney U test, U=843.0, p<0.001), both significant after Bonferroni-correction with an $\alpha = 0.0125$). None of the other ANOVA interactions with *GROUP* were significant (all $p > 0.3$). Further results for the smarties test are shown in **Supplementary Table 5**.

Anatomical lesion mapping

Anatomical data were analysed using the FMRIB software library (FSL 5.0.7, Center for Functional Magnetic Resonance Imaging of the Brain, University of Oxford, UK www.fmrib.ox.ac.uk/fsl). Procedures have been described before [9]. The T1-weighted tFLASH dataset was skull stripped (brain extraction tool BET) and registered to the standard brain template of the Montreal Neurological Institute at a 1 mm isotropic resolution (MNI152,

provided with FSL), using the FMRIB's linear registration tool (FLIRT: 12 parameters affine transformation). The resulting transformation matrix was applied to the whole-head tFLASH dataset (without skull stripping) and the T2-weighted FLAIR dataset as well, after the FLAIR images had been coregistered to the T1 images of the same subject. In a second step, the linearly coregistered, whole-head tFLASH dataset was non-linearly registered to the MNI152 template using the FMRIB's non-linear registration tool (FNIRT). Again, the resulting transformation values were applied to the FLAIR dataset. Finally, FLIRT was used to sample the data to 0.5 mm isotropic resolution using the MNI152 template at 0.5 mm resolution. A digitized version of the Morel atlas of the thalamus [10] provided by Krauth et al. [11], registered to the high-resolution MNI 152 template allowed the visualization of the thalamic nuclei in respect to the thalamic lesions, which were manually segmented on FLAIR MR images using MRIcron and transformed to MNI space (via coregistration of individual FLAIR images to individual T1-weighted images followed by normalization to the MNI152 T1 0.5mm template using FSL FLIRT, FNIRT). Individual scans and results of the lesion mapping are shown in **Supplementary Fig. 2 and Supplementary Table 1.**

Single Patient Descriptions

Individual results of the Optic Ataxia task are shown in **Figure 4** and in **Supplementary Table 3**.

Patient N054: Optic Ataxia without Grasping deficit

N054 is a 70-year old, right handed man who suffered from a left thalamic ischemic stroke. The patient presented with difficulties to find words. The **MRI** showed a lesion in the left medial (MD, CeM, CL, CM) and lateral thalamus (VA, VL, VM, VP). **Neurological examination** at admission showed a modest non-fluent aphasia. The patient's cranial nerve examination showed a facial asymmetry to the right. The reflex-status, muscle tone and strength were normal. **There was no sign of ataxia with a metric finger-to-nose test on both sides.** The experimental tasks and MRI's were performed within the first week after hospital admission due to symptom onset. **Neurophysiological** examinations showed an afferent deficit from both arms and legs as well as an efferent deficit to both legs. **Neuropsychological** assessment revealed normal executive, but abnormal memory and language functions. The Mini Mental Status was lower than average with 24/30 points. Oculomotor functions were normal and visual fields were intact. There was no sign of spatial neglect or extinction. EEG

was normal with an alpha ground rhythm. **Grasping:** N054 showed normal reach-grasp scores in the smarties test with both hands (Grasping CH-CS: z = 0.43, IH-IS: z = 0.80). **Optic Ataxia:** In the optic ataxia task he mostly made corrected or uncorrected errors in the non-foveal condition with the right (i.e. contralesional) hand (corrected: 5.3%, uncorrected: 13.2%). However, the majority of trials (81.6%) with this hand were rated effective and fluent. The slowed and corrected reaches with the right hand in the right space led to a pathological optic ataxia score for the contralesional hand/space in this patient (z = -3.67). The OA scores for the IH-IS condition were in the lower normal range (z = -1.57). While most errors occurred in the combination contralesional hand/field (CH-CS) condition, no other clear hand or space effect can be observed.

Patient N056: Optic Ataxia without Grasping deficit

N056 is a 24-year old, right handed man who suffered from a bilateral (left > right) thalamic hemorrhagic stroke resulting from a sinus venous thrombosis. The patient complained about headaches and fatigue at admission. The **MRI** showed a lesion in the left medial (MD, CL, CM) and lateral thalamus (VL, VP). On the right side, there was a smaller VA damage. **Neurological examination** at admission showed a general slowness as well as a slight paresis and fine motor difficulties of the right arm. Sensibility to touch was intact on both sides. The patient's cranial nerve examination was normal. The reflex-status, muscle tone and other sensory tests were normal. Oculomotor functions were normal and visual fields were intact. There was no sign of spatial neglect or extinction. **There was no sign of ataxia with a metric finger-to-nose test on both sides. Neuropsychological** assessment revealed normal executive, normal memory and normal language functions. The Mini Mental Status was also normal with 30/30 points. The experimental tasks and MRI's were performed within two weeks after hospital admission due to symptom onset. EEG before testing was normal with an alpha ground rhythm. **Grasping:** N056 showed normal reach-grasp scores in the smarties test with both hands (Grasping CH-CS: z = 0.43, IH-IS: z = 0.07). **Optic Ataxia:** In the optic ataxia task he mostly made corrected or uncorrected errors in the non-foveal condition with the right hand (corrected: 16.6%, uncorrected: 11.1%). However, the majority of trials (72.2%) with this hand were rated effective and fluent. The slowed and corrected reaches with the right hand in the right space led to a pathological optic ataxia score for the contralesional hand/space in this patient (z = -3.68). The OA scores for the IH-IS condition were normal. Gross (i.e. uncorrected) reach errors occurred all away from the body and deviated in 4 out of 5 cases

towards the right (i.e. contralesional) side. Most errors occurred in the combination contralesional hand/contralesional field (CH-CS) condition and he made also relatively more errors in the ipsilesional hand/contralesional field (IH-CS) compared to the remaining combinations. There thus seems to be some space effect.

Patient N063: Optic Ataxia with Grasping deficit

N063 is a 50-year old, right handed woman who suffered from a bilateral thalamic ischemic stroke. The patient presented with dysaesthesia, i.e. tingling of the right arm and face. The **MRI** showed a bilateral lesion (left > right) encompassing the medial (CM, CL) and lateral thalamus (bilateral VP, but VL and VM on the left side only), and posterior thalamus (anterior and medial Pulvinar and LP). While the right thalamic lesion was visible in the MRI, it remained clinically silent. Thus for consistency, the left side is used as reference for the label 'contra- or ipsilesional' (i.e. right is contralesional). **Neurological examination** at admission showed a latent paresis on the right side (sinking of the arm). **The finger-to-nose test was slightly dysmetric on the right side.** The patient's cranial nerve examination was normal. The reflex-status, muscle tone and other sensory and muscle strength tests were normal. Oculomotor functions were normal and visual fields were intact. The experimental tasks and MRI's were performed within two weeks after hospital admission due to symptom onset. EEG was normal. **Neuropsychological** exam was conducted on day 8 after admission and revealed normal executive, normal memory and normal language functions. The Mini Mental Status was also normal with 30/30 points. There was no indication of spatial neglect or extinction. **Grasping:** N063 showed pathological reach-grasp scores in the smarties test with the contralesional (right) hand only (z = -6.9). Z-scores for grasping with the ipsilesional hand were normal (z = 0.21). **Optic Ataxia:** In the optic ataxia task she made a large amount of corrected errors in the non-foveal condition with the right hand. Only 30.9% trials with this hand were rated effective and fluent, while the remaining trials were slowed and ineffective but were corrected in flight. The slowed and corrected reaches with the right hand in the right space led to a pathological optic ataxia score in this patient (z = -4.6). The ipsi hand/field OA condition was not impaired (OA z-score: -1.32). Reach errors mainly occurred all away from the body and deviated towards the right (i.e. contralesional) side. While most errors occurred in the combination contralesional hand/field (CH-CS) condition, no other clear hand or space effect could be observed.

Patient N075: Optic Ataxia with minor Grasping deficit

N075 is a 63-year old, right handed man who suffered from a right thalamic ischemic stroke. The patient presented with hypaesthesia of the left cheek and hand. Neurological examination at admission confirmed a brachiofacial hypaesthesia on the left and found a facial asymmetry. The **MRI s**howed a primarily right sided lesion in the right lateral thalamus (VP, VL) with involvement of the medial group (CL, CM, MD (< 10%)) and anterior pulvinar, and in the left thalamus, smaller (possibly older) lesions in the medial (MD, CM, CL), VP and anterior pulvinar. **Neurological examination** showed that apart from the hypaesthesia, the patient's cranial nerve examination was elsewise normal. The reflex-status, muscle tone and other sensory tests were normal. Oculomotor functions were normal and visual fields were intact. There was no sign of spatial neglect or extinction, although he showed a lateralized deficit in the line bisection task. **There was no sign of ataxia with a metric finger-to-nose test on both sides. Neuropsychological assessments** were not performed. The **EEG** showed a normal alpha ground rhythm with intermittent fronto-temporal delta activity on the left side. **Grasping:** N075 showed borderline abnormal reach-grasp scores in the smarties test with the contralesional (left) hand (Grasping CH-CS: z = -1.81, IH-IS: z = -1.1). **Optic Ataxia:** In the optic ataxia task he mostly made corrected or uncorrected errors in the non-foveal condition with the contralesional hand (corrected: 3.6%, uncorrected: 14.29%). However, the majority of trials (82.1%) with this hand were rated effective and fluent. He had a pathological optic ataxia score for the contralesional (left) hand/space (z = -2.81). The OA scores for the IH-IS condition were normal (z = 0.71). Most errors occurred in the combination contralesional hand/contralesional field (CH-CS) condition. He also made relatively more errors in the ipsilesional hand/contralesional field (IH-CS) compared to the remaining conditions. Thus, there seems to be some (contralesional) space effect.

Patient N093 Optic Ataxia with Grasping deficit

N093 is a 71-year old, right handed man who suffered from a right thalamic ischemic stroke. The **MRI** showed a thalamic lesion that included the right medial thalamus (MD, CeM, CMm CL) as well as the lateral thalamus (VL, VM, VP). **Neurological examination** showed a brachiofacial paresis of the left side with a pronation of the left arm and dysarthria. The patient's cranial nerve examination was elsewise normal. The reflex-status, muscle tone and other sensory and muscle strength tests were normal. Sensibility to touch was intact on both sides. Oculomotor functions were normal and visual fields were intact. There was no sign of

spatial neglect or extinction. **There was no sign of ataxia with a metric finger-to-nose test on both sides.** The Mini Mental Status was also normal with 30/30 points. **EEG** was normal with an alpha ground rhythm. **Grasping:** N093 showed impaired grasping in the smarties test with both hands (Grasping CH-CS: z = -3.82, IH-IS: z = 3.39). **Optic Ataxia:** In the optic ataxia task he mostly made corrected errors with his left (contralesional) hand (CH-CS: corrected: 23.3%, uncorrected: 3.3%). There was a clear hand effect, as he showed pathological error scores with the contralesional hand also when the movement was made into the ipsilesional space (CH-CS: corrected: 25.0%, uncorrected: 4.2%). Gross reach errors were mostly away from the body (5 away/ 2 towards) and deviated mostly towards the left side.

Supplementary Figures

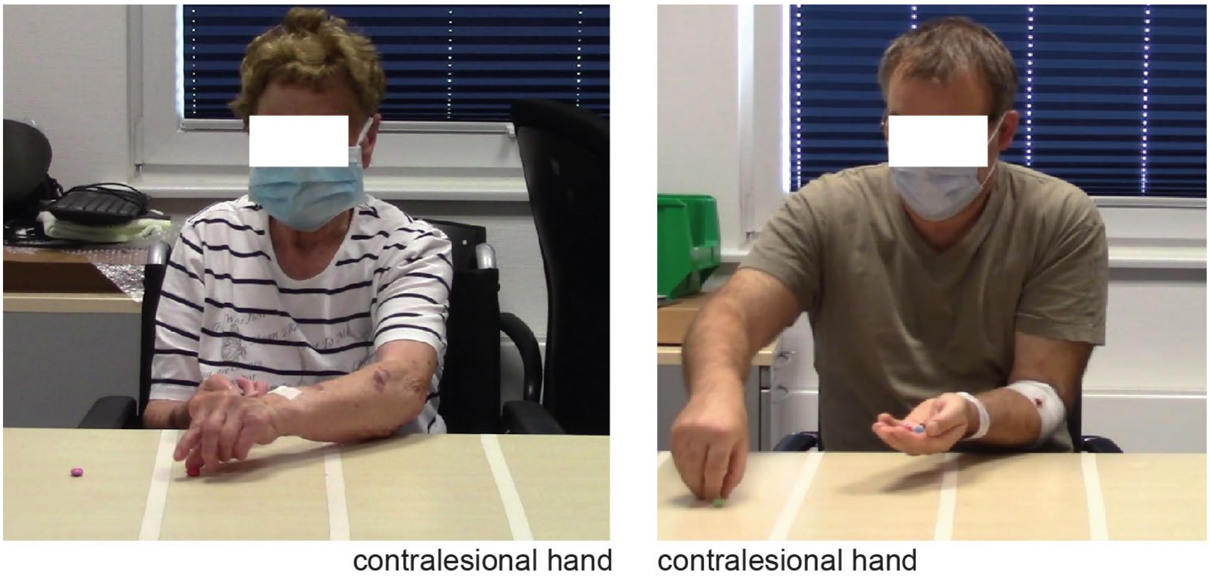

Supplementary Figure 1 Reach-grasp task with small objects (smarties) in two example patients (drawing from video snapshot) with pathological grasp error scores. Typical grasp postures. Left panel: note the abnormal wrist angle and the absence of a precision grip. Right panel: note the grasp deviation from the object location.

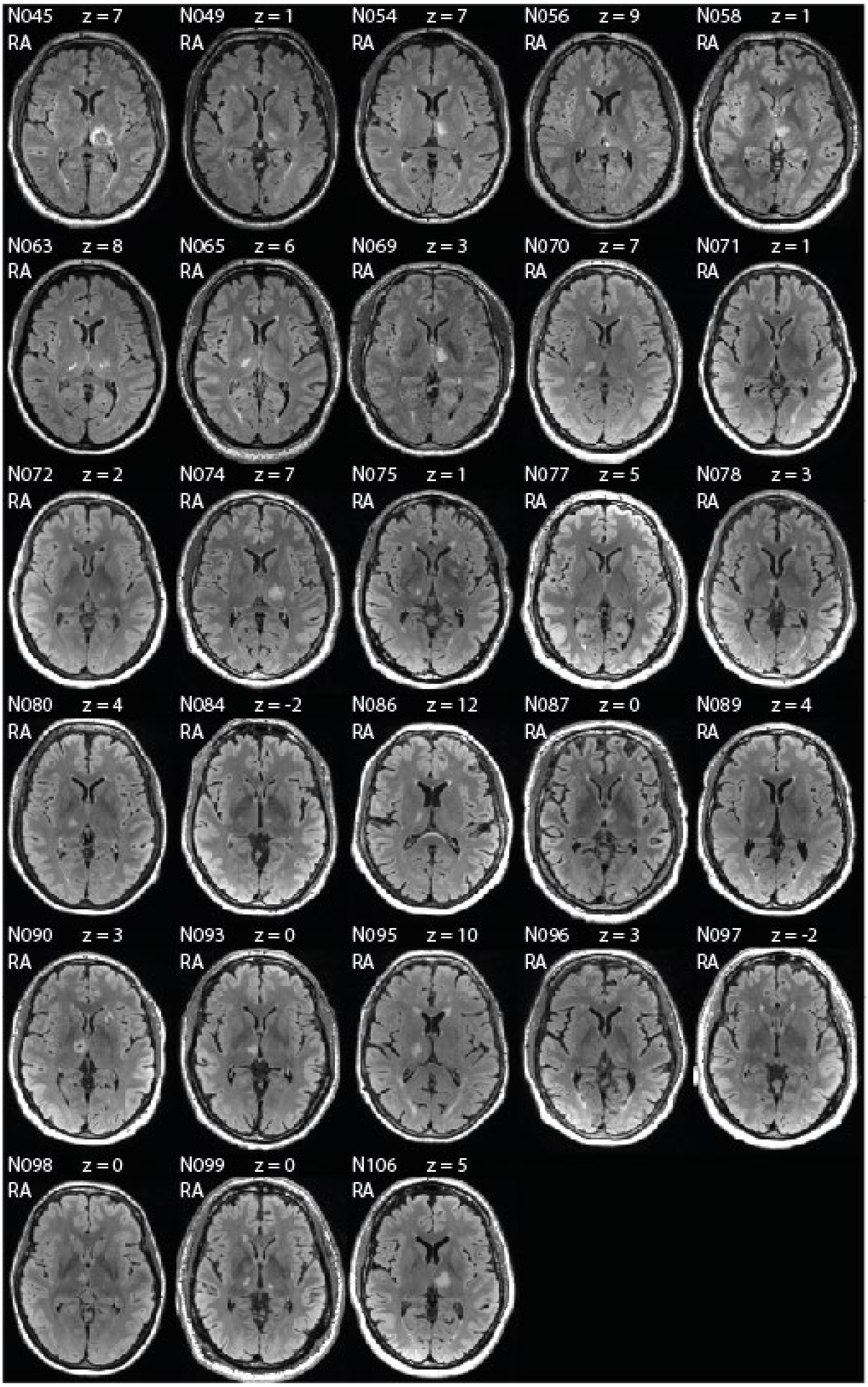

Supplementary Figure 2 Lesion locations. Reconstructed FLAIR axial images showing the lesion location for each patient ($n_{patients}$ = 28). Lesion-centered axial slices are in MNI space, z-coordinates and patient abbreviation (N*) are reported on top of each slide. Slices are oriented in radiological convention: RA = right anterior, on the left.

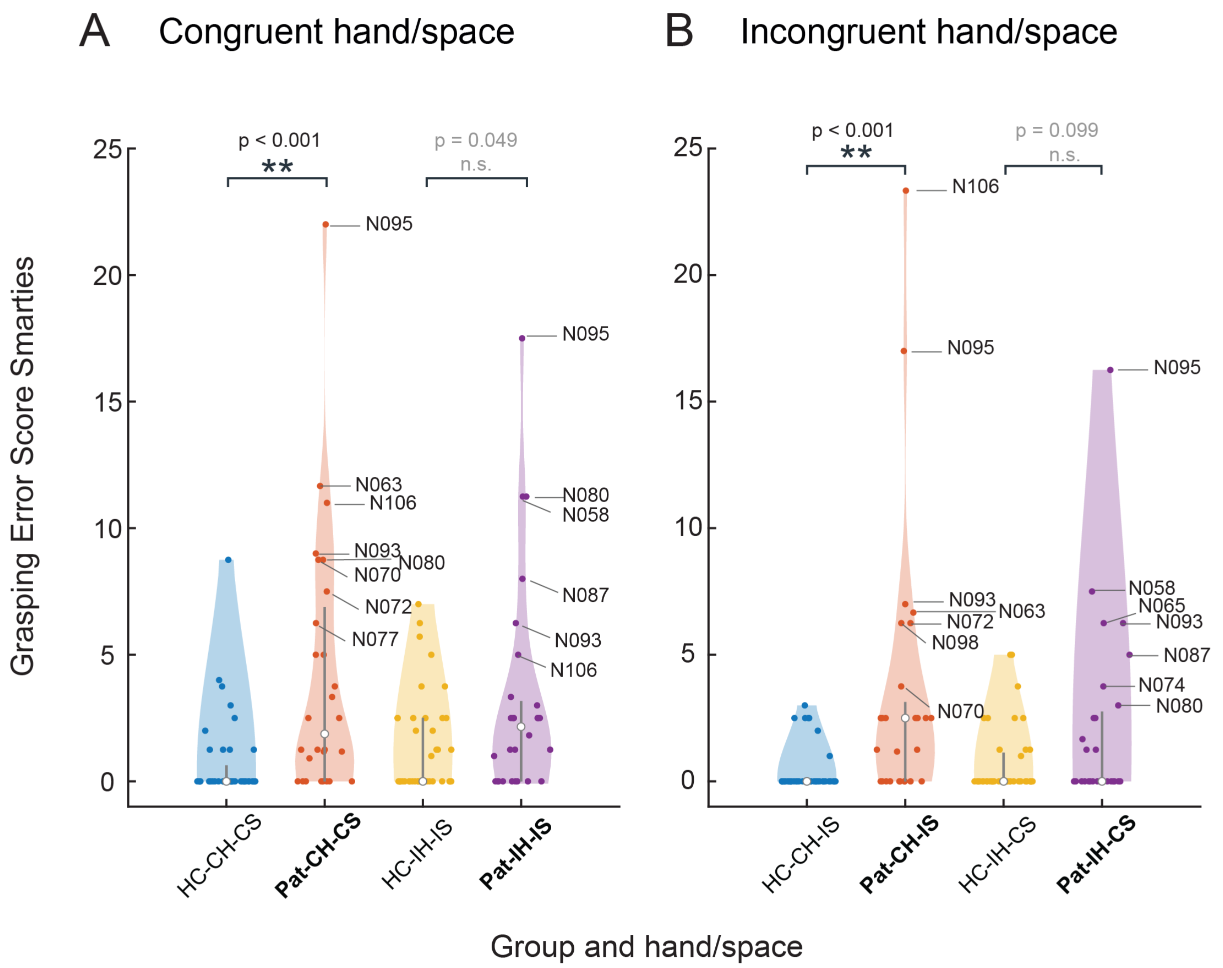

Supplementary Figure 3 Grasping performance of the healthy controls and patients as a function of hand and space ($n_{patients}$ = 28; $n_{healthy}$ = 40). Subjects were always allowed to look at the smarties before and during the reach-grasp. **(A)** Grasping errors across different hand-space conditions of thalamic patients (n = 28) and healthy controls (n = 40). Congruent condition refers to the same hand and space (contralesional hand and space: CH-CS; and ipsilesional hand and space: IH-IS). Patients with thalamic lesions exhibited impaired reach-grasp movements compared to the healthy subjects, more often for the contralesional hand and space (CH-CS: Mann-Whitney U test, U=846.5, p<0.001). This effect was also significant for the ipsilesional hand and space (IH-IS: U=710, p=0.049 (uncorrected)), while not surviving Bonferroni correction for multiple comparisons (4 comparisons HC vs. Pat, corrected threshold would be p = 0.0125.) **(B)** Grasping error scores for the incongruent condition refer to the contralesional hand and ipsilesional space: CH-IS; or ipsilesional hand and contralesional space: IH-CS. The group comparison between healthy subjects and thalamic patients revealed a significant effect for the contralesional hand and ipsilesional space (CH-IS: Mann-Whitney U test, U=843.0, p<0.001), but not for the grasping comparison between HC and patients with the ipsilesional hand and contralesional space (IH-CS: Mann-Whitney U test, U=671.5, p=0.099). The white dot in each violin plot represents the median of the specific subject group and condition, and the gray bar – the IQR. HC refers to the healthy controls and Pat refers to the thalamic patients. The lines towards the dots show the acronym of the patients with a grasping z-score smaller than two standard deviations from the healthy controls (z < -2, also significant in the Crawford-Garthwaite test for single subjects). In (A) and (B), ** refer to p < 0.001.

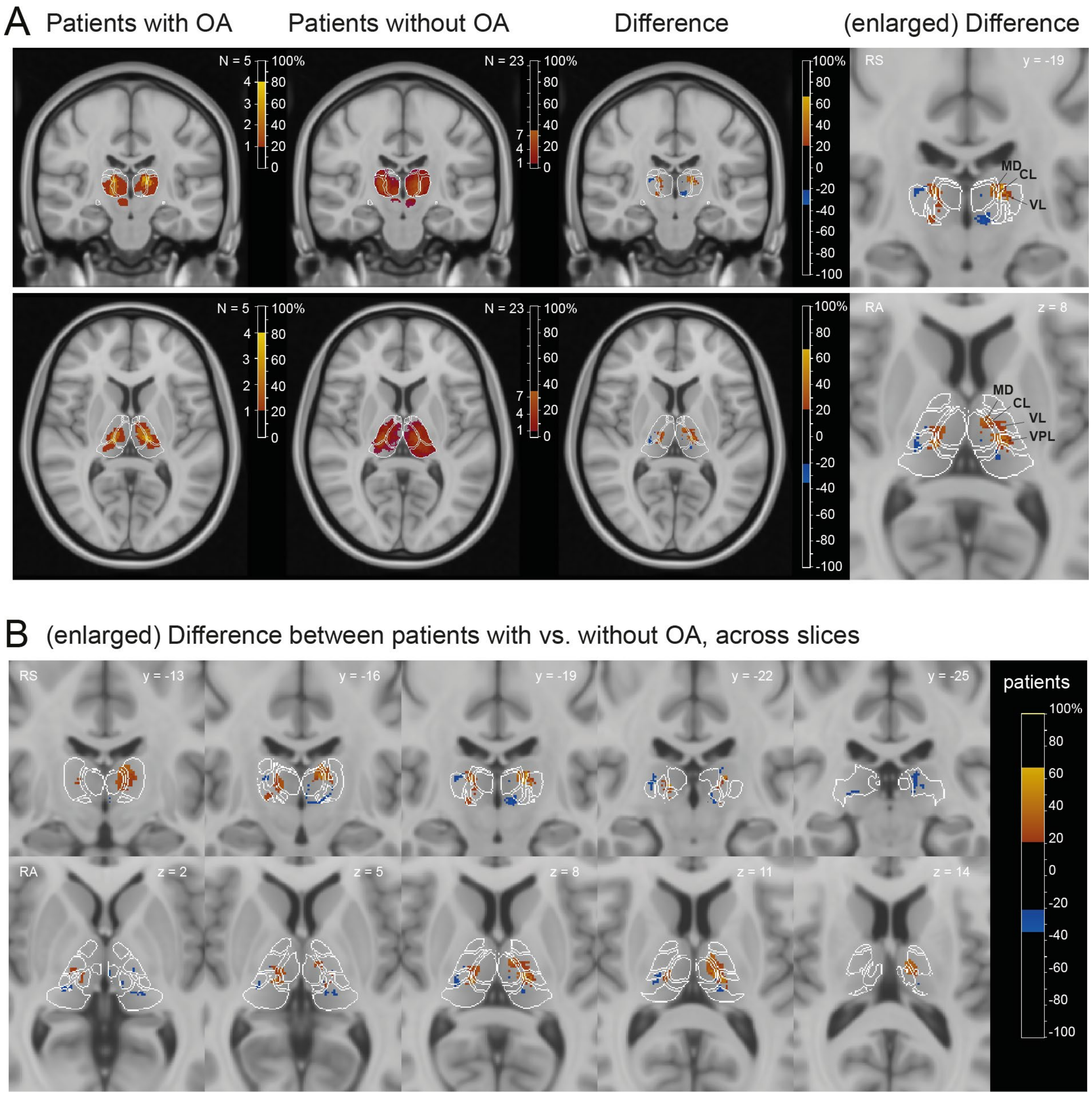

Supplementary Figure 4 Lesion overlap and lesion subtraction plots of the patients with and without optic ataxia in original orientation. (A) Lesion overlap of the patients with ($n_{patients}$ = 5) and without optic ataxia ($n_{patients}$ = 23) (condition: contralesional hand/contralesional space) overlaid onto the MNI template in a coronal (upper row, y=-19) and an axial view (lower row, z=8). Shown are the lesion overlaps of impaired (first column, n=5) and unimpaired patients (second column, n=23) and the lesion subtraction, i.e., the group difference (third column), which was calculated by subtracting the percentage lesioned in the group without optic ataxia from the percentage lesioned in the group with optic ataxia. The heatmap shows the number of patients (N) on the left and the percentage of patients on the right (%). The rightmost column shows the enlarged subtraction plots of these slices. **(B)** Systematic views of the lesion subtraction for optic ataxia across coronal and axial thalamic slices. Positive values in lesion subtraction plots in (A) and (B) in red-yellow indicate stronger lesion load in patients with optic ataxia, whereas negative values in blue-green indicate stronger lesion load in patients without optic ataxia. For visualization purposes group differences were displayed when they exceeded 20%. White outlines represent the borders of selected thalamic nuclei as defined in the digital version of the Morel-atlas normalized to the 0.5 mm MNI152 T1 template. Abbreviations: **VL**, Ventral lateral posterior nucleus; **VPL**, Ventral posterior lateral nucleus; **MD**, Mediodorsal nucleus; **CL**, Central lateral nucleus. Colored schemes of the nuclei outlines are depicted in **Supplementary Figure 5.** In (A) and (B), the numbers refer to the MNI coordinates of the y-axis (coronal slices) and z-axis (axial slices), respectively. Abbreviations: **R**, right; **A**, anterior; **S**, superior. No additional statistics were applied.

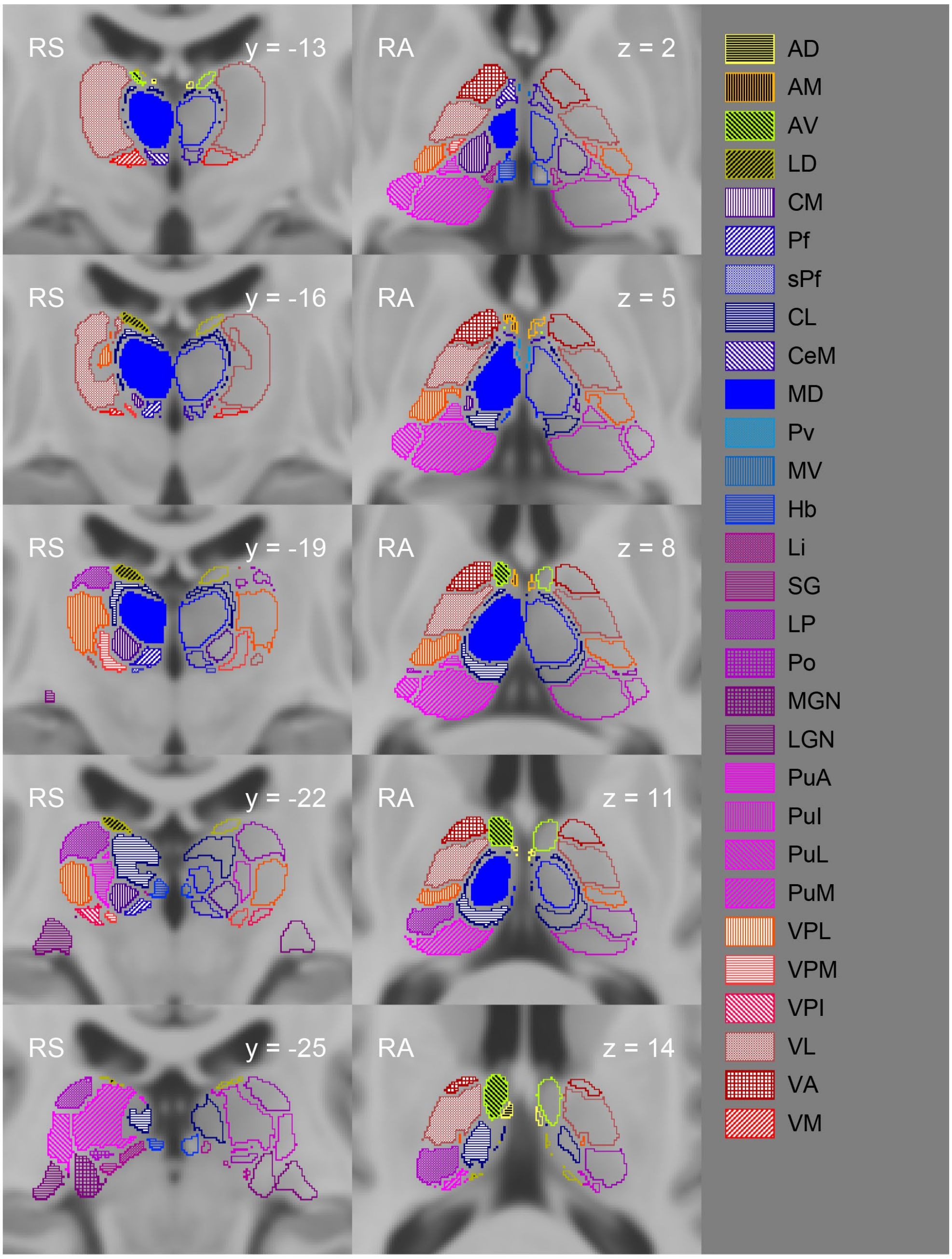

Supplementary Figure 5 Coronal and Axial Slices with thalamic lesion labeling. Abbreviations: **AD**, Anterior dorsal nucleus; **AM**, Anterior medial nucleus; **AV**, Anterior ventral nucleus; **LD**, Lateral dorsal nucleus; **MD**, Mediodorsal nucleus, **CM**, Centromedian nucleus; **Pf**, Parafascicular nucleus; **sPf**, Subparafascicular nucleus; **CL**, Central lateral nucleus; **CeM**, Central medial nucleus; **Pv**, Paraventricular nucleus; **MV**, Medioventral nucleus; **Hb**, Habenular nucleus; **PuA**, Anterior pulvinar; **PuI**, Inferior pulvinar; **PuL**, Lateral pulvinar; **PuM**, Medial pulvinar; **LP**, Lateral posterior nucleus; **Li**, Limitans nucleus; **Po**, Posterior nucleus; **SG**, Suprageniculate nucleus; **LGN**, Lateral geniculate nucleus; **MGN**, Medial geniculate nucleus; **VA**, Ventral anterior nucleus; **VL**, Ventral lateral nucleus; **VM**, Ventral medial nucleus; **VP**, Ventral posterior nucleus (**VPL/VPM/VPI**: lateral/medial/inferior). Thalamic nuclei were classified according to the Morel's neuroanatomical atlas [12,13].

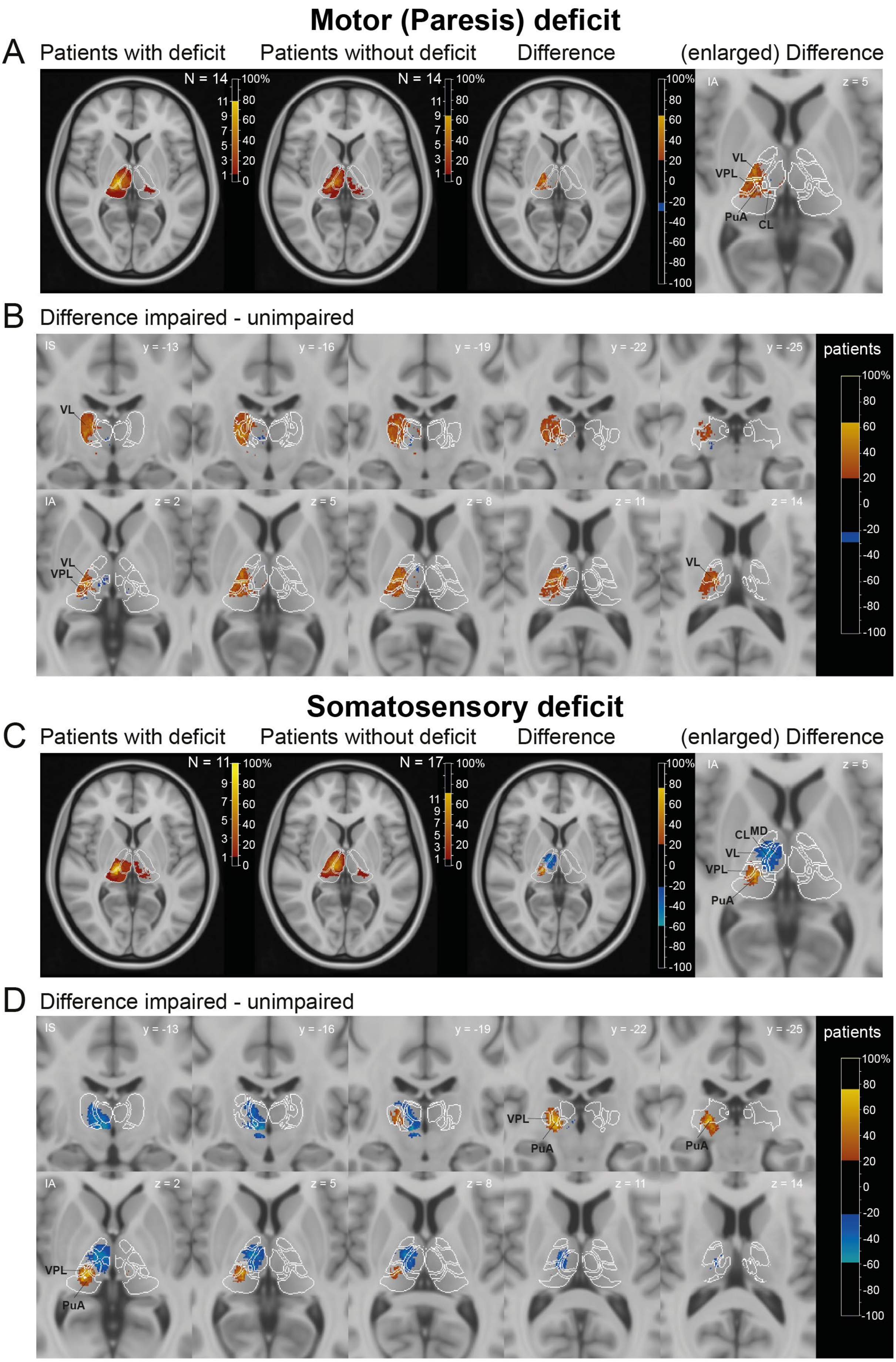

Motor (Paresis) deficit
A
Patients with deficit
Patients without deficit
Difference
(enlarged) Difference
N = 14
N = 14
IA
z = 5
VL
VPL
PuA
CL
B
Difference impaired - unimpaired
IS
y = -13
y = -16
y = -19
y = -22
y = -25
patients
IA
z = 2
z = 5
z = 8
z = 11
z = 14
Somatosensory deficit
C
Patients with deficit
Patients without deficit
Difference
(enlarged) Difference
N = 11
N = 17
CL
MD
D
Difference impaired - unimpaired

Supplementary Figure 6 Control analysis: lesion overlap and lesion subtraction plots of the patients with and without contralesional motor and somatosensory deficits ($n_{patients}$ = 28). (A) Lesion overlap of the patients with ($n_{impaired}$ = 14) and without ($n_{unimpaired}$ = 14) a contralesional upper limb paresis (sinking of the contralesional arm while holding it up). Lesion subtraction plot and thalamic details overlayed on an axial section (z = 5) of the MNI template. **(B)** Systematic views of the lesion subtraction for motor deficits across coronal and axial thalamic slices. Positive values in the lesion subtraction plots in (A) and (B) in red-yellow indicate stronger lesion load in patients with motor deficits, whereas negative values in light to dark blue indicate stronger lesion load in patients without motor deficits. For visualization purposes group differences were displayed when they exceeded 20%. *Upper panel*: coronal, *lower panel:* axial slices **(C)** Lesion overlap and subtraction of the patients with ($n_{impaired}$ = 11) and without ($n_{unimpaired}$ = 17) a contralesional somatosensory deficit. Details as described in (A) **(D)** Systematic views of the lesion subtraction for somatosensory deficits across coronal and axial thalamic slices (as in (B)). White outlines in (A-D) represent the borders of thalamic nuclei as defined in the digital version of the Morel atlas normalized to the 0.5 mm MNI152 T1 template. Colored schemes of nuclei outlines are depicted in **Supplementary Fig. 5.** Abbreviations: **VL**, Ventral lateral nucleus; **VPL**, ventral posterior lateral nucleus; **MD**, Mediodorsal nucleus; **CL**, Central lateral nucleus; **PuA**, Anterior pulvinar. All panels (A-D) show descriptive statistics with numbers of subjects and % of patients with a lesion in a given voxel. In (A-D), the numbers refer to the MNI coordinates of the y-axis (coronal slices) and z-axis (axial slices), respectively. Abbreviations: I, ipsilesional; A, anterior; S superior.

A **Grasping deficit (flipped)**

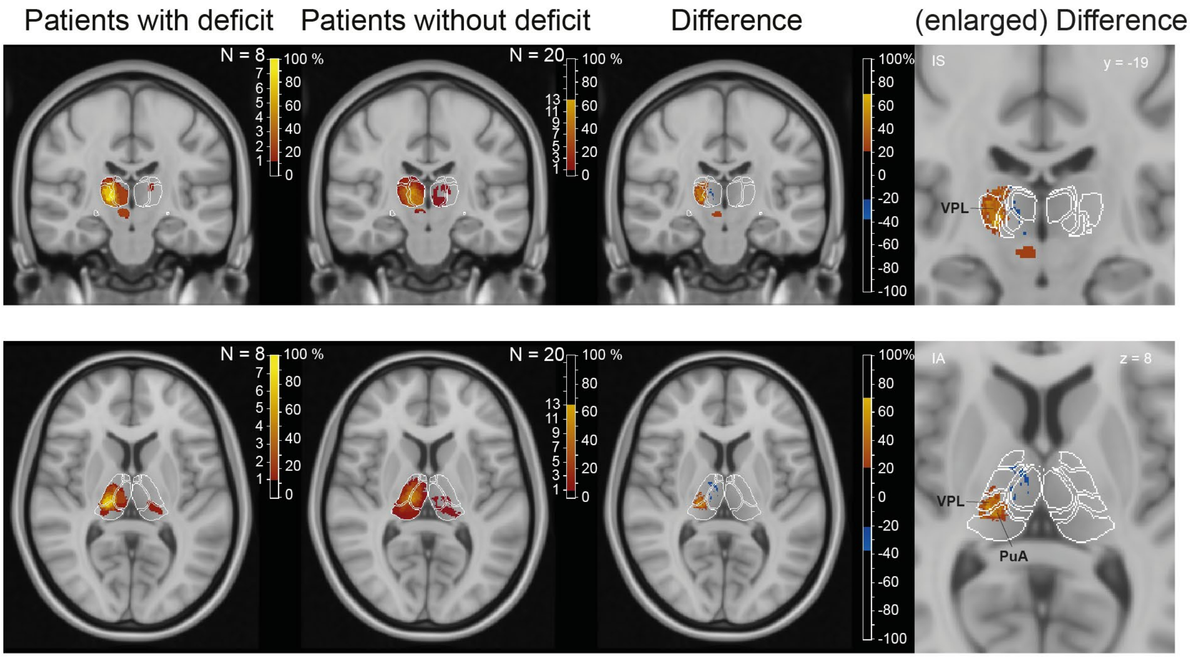

B Difference impaired - unimpaired (flipped)

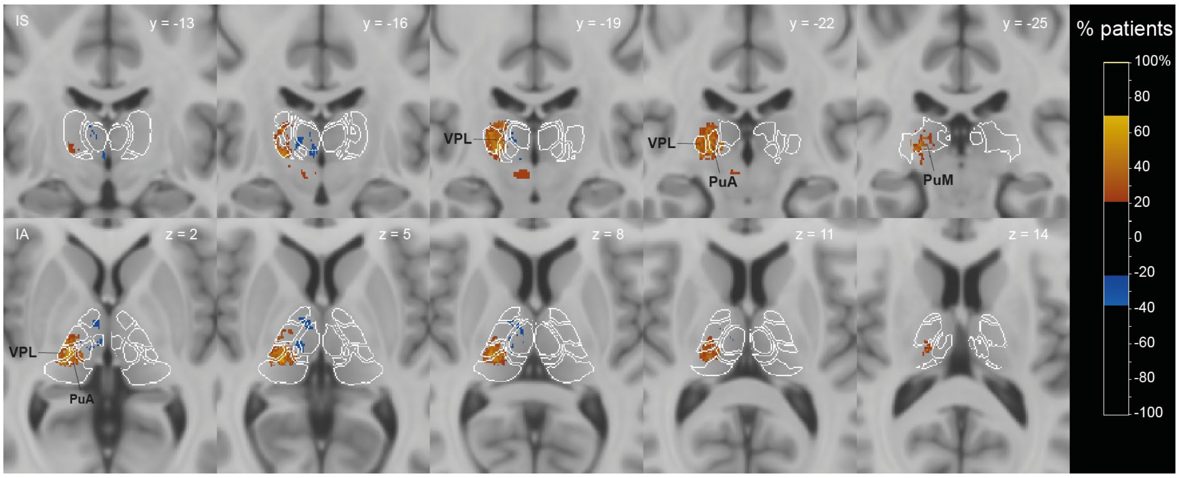

Supplementary Figure 7 Lesion overlap and lesion subtraction plots of the patients with and without contralesional grasping deficits in the smarties task. **(A)** Lesion overlap and lesion subtraction of the patients with ($n_{patients}$ = 8) and without ($n_{patients}$= 20) contralesional grasping deficits, overlaid onto the MNI template in a coronal (upper row, y=-19) and an axial view (lower row, z=8). Primary left thalamic lesions were flipped to the right hemisphere (see **Supplementary Fig. 4** for non-flipped maps). Shown are the lesion overlaps of impaired (first column, n=5) and unimpaired patients (second column, n=23) and the lesion subtraction, i.e., the group difference (third column), which was calculated by subtracting the percentage lesioned in the group without grasping deficits from the percentage lesioned in the group with grasping deficits. The heatmap shows the number of patients (N) on the left and the percentage of patients on the right (%). The rightmost column shows the enlarged subtraction plots of these slices. **(B)** Systematic views of the lesion subtraction for grasping across coronal and axial thalamic slices. Positive values in lesion subtraction plots in (A) and (B) in red-yellow indicate stronger lesion load in patients with grasping deficits, whereas negative values in blue-green indicate stronger lesion load in patients without grasping deficits. For visualization purposes group differences were displayed when they exceeded 20%. White outlines represent the borders of selected thalamic nuclei as defined in the digital version of the Morel-atlas normalized to the 0.5 mm MNI152 T1 template. Abbreviations: **VPL**, ventral posterior lateral nucleus; **MD**, Mediodorsal nucleus; **CL**, Central lateral nucleus; **PuA**, Anterior pulvinar. Thalamic nuclei on selected coronal and axial slices are depicted in **Supplementary Fig. 5**. In (A) and (B), the numbers refer to the MNI coordinates of the y-axis (coronal slices) and z-axis (axial slices), respectively. Abbreviations: I, ipsilesional for flipped; A, anterior S, superior.

Supplementary Tables

Supplementary Table 1 Distribution of lesion sites of each patient

	Anterior				Medial									Posterior										Lateral			
Patient	AD	AM	AV	LD	MD	CM	Pf	sPf	CL	CeM	Pv	MV	Hb	PuA	Pul	PuL	PuM	LP	Li	Po	SG	LGN	MGN	VA	VL	VM	VP
N045L	++		+	+++	+++	+++	+++	+	+++	+			+++	+++		++	+++	+++	+++	+++	+++		+		++	+	+++
N049L						++	+							++			+		+	+++	++		+				+
N054L			+		++	+	+	+	++	++	+	+	+	+				+						+	++	++	+
N056L			+	+	+	+			+																+		+
N056R																								+			
N058L					++	+++	+++	+++	+	+		+	++	+					+						+	+++	+
N063L						++			+					+++	+	+	+	++		++	+	+	++		+	+	+++
N063R						+			+					++		+	+	+									+
N065R					+	+			+					+++		+	+	+		+			+		+		++
N069L		+	+		++	+	+	+++	+	+++		+++		+										++	++	+++	+
N070R					+	+			+					+++		+	+	++	+	++	+		+		+		++
N071R					+	+	++	++					+						+								
N072L						+								++			+			+++	++		++				+
N074L				+	+	++			++					+++		++	+	+++		+			+		++	+	+++
N075L					+	+			+					++			+	+	+	++	+						+
N075R					+	++			+					+++						+					+		++
N077L														++		+	+	+		+			+				++
N078R		+			+	+	+++	++	+	+++	++	+++	+						+					+	+	+	
N080R						++			+					+++			+	+		+			+		+	+	+++
N084L					+	++	+	+++	+	+				+											+	+	+
N086R									+									+						+	++		+
N087L					+	+	++	+++	+	++		+++												++	+	+++	+
N089R						+			+					+++		+	+	+		+			+				++
N090L												+															
N090R					++	+++	+++	+++	++	+++	+	+++	+	+					+	+				++	++	+++	+
N093R					++	+++	+++	+++	+	+		+	+	+					+					+	+	+++	+
N095R				+	+	+			+					++		+	+	+++		+			+		++	+	+++
N096L				+	++	+++	+++	++	++	+		+	+	+++		+	+++	++	+++	+++	+++		+				+
N096R					+	++	+++	++	+	+			+				+		++		+						+
N097R						+	+							+					+	++	++		+				+
N098R					+	++	+++	+++	+	+		+	+						+						+	+++	+
N099L						+	+		+					+		+	+	+	+	++	++		+				
N099R						+								+++			+	+	+	+++	++		+		+	+	++
N106L					++	+++	+++	+++	++	+		+	+++	+++			+	+	+++	++	+			+	++	+++	+++

This table delineates the thalamic nuclei impacted by the lesion, classified according to the Morel's neuroanatomical Atlas (2007). The extent of the lesion is presented for each nucleus, denoted as follows: "+" indicates less than 1/3 volume affected, "++" signifies between 1/3 and 2/3 volume affected, and "+++" denotes more than 2/3 of the nucleus volume affected. Abbreviations: **AD**, Anterior dorsal nucleus; **AM**, Anterior medial nucleus; **AV**, Anterior ventral nucleus; **LD**, Lateral dorsal nucleus; **MD**, Mediodorsal nucleus, **CM**, Centromedian nucleus; **Pf**, Parafascicular nucleus; **sPf**, Subparafascicular nucleus; **CL**, Central lateral nucleus; **CeM**, Central medial nucleus; **Pv**, Paraventricular nucleus; **MV**, Medioventral nucleus; **Hb**, Habenular nucleus; **PuA**, Anterior pulvinar; **PuI**, Inferior pulvinar; **PuL**, Lateral pulvinar; **PuM**, Medial pulvinar; **LP**, Lateral posterior nucleus; **Li**, Limitans nucleus; **Po**, Posterior nucleus; **SG**, Suprageniculate nucleus; **LGN**, Lateral geniculate nucleus; **MGN**, Medial geniculate nucleus; **VA**, Ventral anterior nucleus; **VL**, Ventral lateral nucleus; **VM**, Ventral medial nucleus; **VP**, Ventral posterior nucleus

Supplementary Table 2 Frequency of thalamic lesion sites ($n_{patients}$ = 28)

Number of Patients	Patients in %	Nucleus
23	82.14	CM
20	71.42	VP
18	64.28	PuA
15	53.57	VL
14	50	CL
13	46.42	Pf
13	46.42	Po
13	46.42	sPf
11	39.28	LP
11	39.28	MD
11	39.28	VM
10	35.71	CeM
8	28.57	Hb
8	28.57	Li
8	28.57	MGN
8	28.57	SG
7	25	MV
6	21.42	VA
4	14.28	PuL
3	10.71	PuM
2	7.14	LD
2	7.14	Pv
1	3.57	AD
1	3.57	AM
0	0	AV
0	0	LGN
0	0	PuI

In case of bilateral lesions the largest lesion site was counted. The minimum lesion size was set to 10% of the nucleus. Abbreviations: **AD**, Anterior dorsal nucleus; **AM**, Anterior medial nucleus; **AV**, Anterior ventral nucleus; **CM**, Centromedian nucleus; **LD**, Lateral dorsal nucleus; **LGN**, Lateral geniculate nucleus; **LP**, Lateral posterior nucleus; **Li**, Limitans nucleus; **MD**, Mediodorsal nucleus; **MGN**, Medial geniculate nucleus; **MV**, Medioventral nucleus; **Pf**, Parafascicular nucleus; **Po**, Posterior nucleus; **CL**, Central lateral nucleus; **CeM**, Central medial nucleus; **Pv**, Paraventricular nucleus; **Hb**, Habenular nucleus; **PuA**, Anterior pulvinar; **PuI**, Inferior pulvinar; **PuL**, Lateral pulvinar; **PuM**, Medial pulvinar; **SG**, Suprageniculate nucleus; **sPf**, Subparafascicular nucleus; **VA**, Ventral anterior nucleus; **VL**, Ventral lateral nucleus; **VM**, Ventral medial nucleus; **VP**, Ventral posterior nucleus

Supplementary Table 3 Demographic, Clinical and Experimental data for each patient

	Gender	Age	Handedness	Etiology	Side	Lesion size	Optic Ataxia contra	Somatosensory upper contra	Somatosensory lower contra	Paresis upper contra	Paresis lower contra	CL fMAS contra	Neglect Present	Ataxia contra	Ataxia ipsi	MMSE/MOCA	Z Score SM Error CH CS Fov	Z Score SM Error IH IS Fov	Z Score AT Error CH CS	Z Score AT Error IH IS	OA z CH CS	OA z IH IS
**N063**	f	50	2	1	1	1317	1	0	0	1	1	0	0	1	0	30	-6.70	-0.21	-5.65	-1.53	-4.61	-1.32
**N056**	m	25	2	2	1	759	1	0	0	1	0	0	0	0	0		0.43	0.07	0.60	0.78	-3.68	-0.06
**N093**	m	71	2	1	2	1779	1	0	0	1	0	1	0	0	0		-3.82	-3.39	0.37	-0.70	-3.31	1.03
**N054**	m	69	2	1	1	1947	1	0	0	0	0	1	0	0	0	24	0.43	-0.80	0.60	-0.52	-3.07	-1.57
**N075**	m	63	2	1	2	899	1	1	0	0	0	0	0	0	0		-1.81	-1.10	-0.09	-2.81	-2.83	0.71
**N058**	m	32	2	1	1	1214	0	0	0	0	0	0	0	0	0		-0.33	-4.95	0.60	0.78	-1.49	-2.24
**N080**	f	62	2	1	2	1088	0	1	1	1	1	0	0	0	1	30	-3.69	-6.45	-0.45	0.60	-1.31	0.48
**N097**	m	56	2	1	2	171	0	1	0	0	0	0	0	0	0	30	0.70	0.43	0.78	0.60	-1.13	0.48
**N078**	m	77	2	1	2	1185	0	0	0	0	0	1	0	0	0	28	0.07	0.43	-0.52	-0.62	-1.11	-1.34
**N072**	f	57	2	1	1	384	0	1	1	1	1	0	0	0	0	30	-4.15	0.20	-1.67	0.35	-1.01	0.41
**N098**	f	55	2	1	2	847	0	0	0	0	0	1	0	0	0	23	0.70	-1.61	0.78	0.60	-0.59	0.12
**N077**	m	50	2	1	1	533	0	1	1	0	0	0	0	0	0		-3.39	-0.55	-0.54	-0.77	-0.55	-5.58
**N074**	m	51	2	1	1	2797	0	0	0	1	1	0	0	1	0		-1.10	-0.55	0.60	0.39	-0.51	0.43
**N106**	m	52	2	1	1	3438	0	0	0	1	1	1	0	0	0	29	-6.29	-1.81	-6.67	0.78	-0.47	0.48
**N065**	m	53	2	1	2	822	0	1	1	1	0	0	0	0	0	29	0.70	-1.10	-1.53	-0.62	-0.35	0.19
**N069**	m	48	2	1	1	1992	0	0	0	0	0	1	0	0	0	29	0.43	0.70	0.03	0.78	-0.28	0.21
**N071**	f	76	2	1	2	192	0	0	0	0	0	0	0	0	0	29	-1.81	-0.33	0.35	0.60	-0.17	-0.12
**N087**	m	52	2	1	1	805	0	0	0	1	0	1	1	0	0	25	-0.12	-3.32	0.60	0.78	-0.12	0.48
**N090**	m	51	2	1	2	2613	0	0	0	1	1	0	0	0	0	27	0.07	0.43	0.78	0.60	0.03	0.10
**N099**	m	60	2	1	2	959	0	1	0	0	0	0	0	0	0		0.11	0.43	0.78	0.60	0.06	0.48
**N084**	m	61	2	1	1	367	0	0	0	0	0	0	0	0	0	29	0.43	0.70	0.60	0.78	0.08	0.21
**N070**	m	59	2	1	2	1384	0	0	0	0	0	0	0	1	0	29	-3.69	-0.33	-0.09	0.60	0.11	-0.12
**N086**	f	71	3	1	2	1069	0	0	0	1	1	1	0	1	0	29	-1.18	0.43	-0.52	0.60	0.33	-1.88
**N096**	m	71	2	1	1	2850	0	1	1	0	0	0	0	0	0	22	0.43	0.70	0.60	0.78	0.47	0.48
**N089**	m	67	2	1	2	653	0	1	1	1	0	0	0	0	0	29	-0.55	-1.10	-0.04	0.60	0.62	0.48
**N095**	f	80	2	1	2	2156	0	0	0	1	1	0	0	0	0		-10.35	-10.27	-11.78	-4.59	0.95	-0.35
**N049**	m	70	2	1	1	438	0	1	0	0	0	0	0	0	0	28	-1.61	-0.55	-1.67	-1.68	1.18	-0.43
**N045**	m	59	2	2	1	5713	0	1	1	1	1	0	0	0	0		-0.29	0.70	-1.22	0.78	1.22	0.48

Abbreviations: **Z Score_SM Error CH CS_Fov**, Z-Score of the grasping errors with the contralesional hand into the contralesional space; **Z Score_SM Error IH IS Fov**, Z-Score of the grasping errors with the ipsilesional hand into the ipsilesional space; **Z_Score_AT_Error_CH CS**, Z-Score of reach-grasp errors in the Optic Ataxia task with the contralesional hand in contralesional space; **Z_Score_AT_Error_IH IS**, Z-Score of reach-grasp errors in the Optic Ataxia task with the ipsilesional hand in ipsilesional space; **OA z CH CS**, z-score of Optic Ataxia Scores for reaches with the contralesional hand into the contralesional space (error score in the peripheral condition - error score in the peripheral condition); **OA z IH IS**, z-score of Optic Ataxia Scores for reaches with the ipsilesional hand into the ipsilesional space (error score in the peripheral condition - error score in the peripheral condition). **MMSE**, The Mini Mental State Examination; **MoCA**, Montral Cognitive Assessment. Patients were sorted according to their **OA z CH CS** scores. Empty cells correspond to non-existing data.

Supplementary Table 4 Reach error scores and optic ataxia scores for patient groups and healthy controls

	Thalamus Patients	Healthy Controls
AT_CH_CS_Fov	0.9 [0.0 17.0]	0.0 [0.0 4.7]
AT_CH_CS_nonFov	3.4 [0.0 20.5]	1.2 [0.0 12.1]
AT_IH_IS_Fov	0.9 [0.0 5.7]	0.0 [0.0 5.0]
AT_IH_IS_nonFov	3.4 [0.0 18.0]	1.2 [0.0 10.0]
AT_CH_IS_Fov	0.0 [0.0 16.2]	0.0 [0.0 3.8]
AT_CH_IS_nonFov	0.0 [0.0 16.2]	0.0 [0.0 3.8]
AT_IH_CS_Fov	0.0 [0.0 16.2]	0.0 [0.0 3.8]
AT_IH_CS_nonFov	1.0 [0.0 16.2]	0.0 [0.0 3.8]
OA_Score_CH_CS	2.4 [-2.0 13.6]	0.5 [-4.7 10.4]
OA_Score_IH_IS	0.7 [-1.4 15.9]	0.0 [-3.1 7.6]
OA_AtaxiaScore_CH_IS	0.0 [-12.2 19.4]	0.0 [-2.7 5.9]
OA_AtaxiaScore_IH_CS	0.0 [-2.3 7.2]	0.0 [-2.9 7.6]
AT_PercError_CH_CS_Fov	3.0 [0.0 55.0]	0.0 [0.0 15.6]
AT_PercError_CH_CS_nonFov	10.0 [0.0 69.0]	0.0 [0.0 25.0]
AT_PercError_IH_IS_Fov	0.0 [0.0 16.7]	2.5 [0.0 25.0]
AT_PercError_IH_IS_nonFov	3.7 [0.0 39.3]	0.0 [0.0 25.0]
AT_PercError_CH_IS_Fov	0.0 [0.0 42.9]	0.0 [0.0 11.5]
AT_PercError_CH_IS_nonFov	0.0 [0.0 54.5]	0.0 [0.0 13.6]
AT_PercError_IH_CS_Fov	0.0 [0.0 42.9]	0.0 [0.0 11.5]
AT_PercError_IH_CS_nonFov	2.1 [0.0 20.0]	0.0 [0.0 16.7]
AT_UnCorPercError_CH_CS_Fov	0.0 [0.0 2.5]	0.0 [0.0 4.5]
AT_UnCorPercError_CH_CS_nonFov	0.0 [0.0 14.3]	0.0 [0.0 7.1]
AT_UnCorPercError_IH_IS_Fov	0.0 [0.0 3.6]	0.0 [0.0 5.9]
AT_UnCorPercError_IH_IS_nonFov	0.0 [0.0 13.3]	0.0 [0.0 10.7]
AT_UnCorPercError_CH_IS_Fov	0.0 [0.0 19.2]	0.0 [0.0 3.8]
AT_UnCorPercError_CH_IS_nonFov	0.0 [0.0 18.2]	0.0 [0.0 8.3]
AT_UnCorPercError_IH_CS_Fov	0.0 [0.0 19.2]	0.0 [0.0 3.8]
AT_UnCorPercError_IH_CS_nonFov	0.0 [0.0 8.3]	0.0 [0.0 6.7]

The median and the range are shown. AT_* signifies the reach error scores. Same hand and space conditions ('congruent'): contralesional hand and space: AT_CH-CS and ipsilesional hand and space: AT_IH-IS). Differing hand and space ('incongruent'): ipsilesional hand and contralesional space: AT_IH_CS, contralesional hand and ipsilesional space: AT_CH_IS. The _fov indicates that patients look at the pen while reaching and _nonFov indicates the peripheral conditions, when subjects fixated the camera. OA_ stands for the optic ataxia score as a function of hand and space. Percentage of corrected and uncorrected reach errors. AT_PercError_* includes the 'fluent' (score: 0) and 'corrected' (slowed, insecure and corrected during reach, score: 1-2) and AT_UnCorPercError_'uncorrected_*' includes the (scores 3-5), i.e. when the hand stopped at the wrong position and was corrected only in later reaches.

Supplementary Table 5 Median and range of reach-grasp errors with small objects ("smarties")

	Contra hand, contra space	Ipsi hand, Ipsi space	Contra hand, ipsi space	Ipsi hand, contra space
**Thalamus Patients**	1.9 [0.0, 22.0]	2.2 [0.0, 17.5]	2.5 [0.0, 23.3]	0.0 [0.0, 16.3]
**with left hemisphere lesion**	1.2 [0.0, 11.7]	2.2 [0.0, 11.3]	1.8 [0.0, 23.3]	0.6 [0.0, 7.5]
**with right hemisphere lesion**	3.1 [0.0, 22.0]	1.9 [0.0, 17.5]	2.5 [0.0, 17.0]	0.0 [0.0, 16.3]
**Healthy Controls**	0.0 [0.0, 8.8]	0.0 [0.0, 7.0]	0.0 [0.0, 3.0]	0.0 [0.0, 5.0]

Supplementary References

1. Fels M, Geissner E. Neglect-Test (NET): ein Verfahren zur Erfassung visueller Neglectphänomene; Handanweisung; deutsche überarbeitete Adaption des Behavioural Inattention Test (Wilson, Cockburn & Halligan, 1987). Published online 1997.

2. Bickerton WL, Samson D, Williamson J, Humphreys GW. Separating forms of neglect using the Apples Test: validation and functional prediction in chronic and acute stroke. *Neuropsychology*. 2011;25(5):567-580. doi:10.1037/a0023501

3. Rorden C, Karnath HO. A simple measure of neglect severity. *Neuropsychologia*. 2010;48(9):2758-2763. doi:10.1016/j.neuropsychologia.2010.04.018

4. Vaes N, Lafosse C, Nys G, et al. Capturing peripersonal spatial neglect: an electronic method to quantify visuospatial processes. *Behav Res Methods*. 2015;47(1):27-44. doi:10.3758/s13428-014-0448-0

5. Posner MI, Snyder CR, Davidson BJ. Attention and the detection of signals. *J Exp Psychol*. 1980;109(2):160-174.

6. Rengachary J, He BJ, Shulman GL, Corbetta M. A behavioral analysis of spatial neglect and its recovery after stroke. *Front Hum Neurosci*. 2011;5:29. doi:10.3389/fnhum.2011.00029

7. Rengachary J, d'Avossa G, Sapir A, Shulman GL, Corbetta M. Is the posner reaction time test more accurate than clinical tests in detecting left neglect in acute and chronic stroke? *Arch Phys Med Rehabil*. 2009;90(12):2081-2088. doi:10.1016/j.apmr.2009.07.014

8. Wilke M, Turchi J, Smith K, Mishkin M, Leopold DA. Pulvinar Inactivation Disrupts Selection of Movement Plans. *Journal of Neuroscience*. 2010;30(25):8650-8659. doi:10.1523/JNEUROSCI.0953-10.2010

9. Wilke M, Schneider L, Dominguez-Vargas AU, et al. Reach and grasp deficits following damage to the dorsal pulvinar. *Cortex*. 2018;99:135-149. doi:10.1016/j.cortex.2017.10.011

10. Morel A, Magnin M, Jeanmonod D. Multiarchitectonic and stereotactic atlas of the human thalamus. *J Comp Neurol*. 1997;387(4):588-630. doi:10.1002/(sici)1096-9861(19971103)387:4<588::aid-cne8>3.0.co;2-z

11. Krauth A, Blanc R, Poveda A, Jeanmonod D, Morel A, Székely G. A mean three-dimensional atlas of the human thalamus: generation from multiple histological data. *Neuroimage*. 2010;49(3):2053-2062. doi:10.1016/j.neuroimage.2009.10.042

12. Krauth A, Blanc R, Poveda A, Jeanmonod D, Morel A, Székely G. A mean three-dimensional atlas of the human thalamus: generation from multiple histological data. *Neuroimage*. 2010;49(3):2053-2062. doi:10.1016/j.neuroimage.2009.10.042

13. Morel A, Magnin M, Jeanmonod D. Multiarchitectonic and stereotactic atlas of the human thalamus. *J Comp Neurol*. 1997;387(4):588-630. doi:10.1002/(sici)1096-9861(19971103)387:4<588::aid-cne8>3.0.co;2-z
